# Post-synaptic facilitation and network dynamics underlying stimulus-specific combination sensitivity

**DOI:** 10.1016/j.isci.2026.116125

**Published:** 2026-05-27

**Authors:** Zeina Merabi, Arij Daou

**Affiliations:** 1 Neurophysiology and Computational Neuroscience Group, Biomedical Engineering Program, American University of Beirut, Beirut, Lebanon

**Keywords:** neuroscience, sensory neuroscience, systems neuroscience

## Abstract

Combination-sensitive neurons (CSNs) transform specific combinations of sensory features into selective neural responses, supporting the temporal organization of complex signals. We developed a biophysically realistic computational model, constrained by intrinsic and synaptic properties, to examine how temporally ordered stimulus pairs can be associated across delays of hundreds of milliseconds. The model shows that upstream inhibitory-delay circuits can transiently preserve information about the first stimulus while progressively shaping the excitability of an output neuron. Together with post-inhibitory rebound and facilitation, these dynamics create a delay-dependent primed state that funnels separated inputs into a narrow coincidence-detection window. This mechanism enables sharply timed, selective responses to stimulus pairs without requiring sustained spiking in the output neuron. Our results provide a circuit-level link between coincidence detection and longer-timescale temporal integration, offering a general framework for temporal coding in sensory systems.

## Highlights

• Layered inhibition bridges stimulus pairs across long temporal delays
• Delay-dependent priming creates a narrow coincidence-detection window
• Rebound currents and circuit timing shape selective facilitated bursting
• Temporal order, delay, and input features determine facilitation

## Graphical abstract

**Figure:**
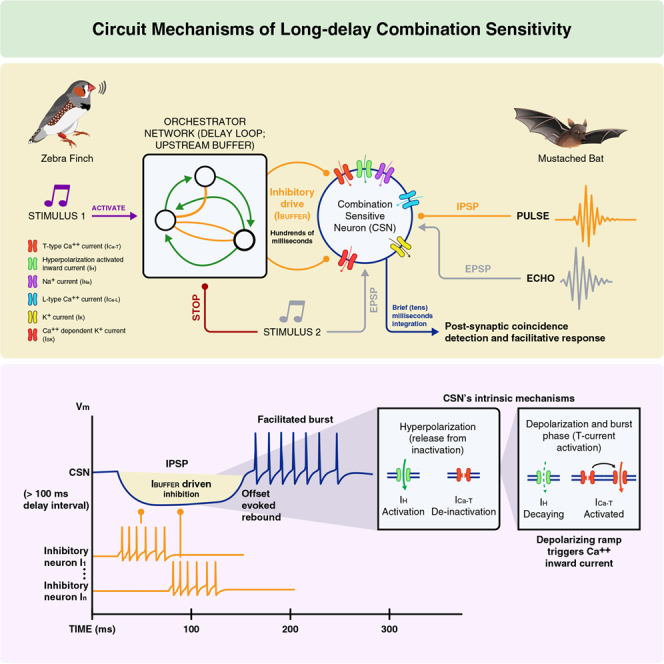

## Introduction

Sensory systems do not simply transmit information from the periphery to the brain; rather, they actively transform incoming signals to create meaningful representations of the external world. This transformation is essential for guiding behavior, allowing organisms to detect, interpret, and respond to complex environmental stimuli. A key aspect of this process is the extraction of behaviorally relevant information from peripheral inputs.^1^ As sensory information ascends through processing hierarchies, neurons often exhibit selectivity for specific combinations of stimulus features.^2^^,^^3^ These so-called combination-sensitive neurons (CSNs) provide unique information that cannot be captured by single stimulus features alone.^4^^,^^5^^,^^6^^,^^7^ For example, the visual system integrates attributes like shape, size, color, and motion, and certain cortical neurons display striking selectivity for complex stimuli such as faces or objects.^8^^,^^9^^,^^10^

Auditory systems also exhibit CSNs across species, including frogs,^11^^,^^12^ songbirds,^4^^,^^5^^,^^13^^,^^14^^,^^15^^,^^16^^,^^17^^,^^18^^,^^19^^,^^20^ bats,^21^^,^^22^ mice,^23^ cats,^24^ and primates.^25^ In bats, delay-tuned neurons integrate pulse-echo pairs over millisecond timescales, serving as coincidence detectors crucial for echolocation.^3^ Unlike bats, songbirds exhibit syllable-specific CSNs in HVC that integrate acoustic information over hundreds of milliseconds, enabling complex vocal communication and song discrimination.^4^^,^^15^^,^^18^^,^^26^ In bats, combination-sensitive behavior involves integration over ∼4 ms in delay-tuned neurons^3^^,^^27^ and ∼20 ms for duration-tuned neurons,^28^ while in songbirds this behavior extends over several hundred milliseconds (∼235 ± 73 ms) for a pair of syllables.^4^

The extended temporal integration required in songbirds raises important questions: how do neurons preserve information across syllables, associate inputs with high temporal precision, and generate appropriate behavioral responses? In particular, how do syllable-specific CSNs manage to maintain the information from the first syllable intact,^29^ prepare for the release and active association with the second syllable in an extremely precise manner over extended durations^4^ and successfully generate an appropriately guiding behavior? One view is that such processing arises not solely from single-cell mechanisms but from dynamic, network-level transformations of neural activity over time.^30^^,^^31^

Building on this perspective, we developed a time-dependent computational model to test how intrinsic neuronal properties and network dynamics contribute to extended temporal integration in combination-sensitive circuits. While our current investigations focus on songbirds, we contend that combination sensitivity is a ubiquitous neural mechanism with broad implications, applicable across diverse species and sensory systems where it plays a crucial role. Our results show how intrinsic neuronal properties, synaptic dynamics, and upstream circuit mechanisms can endow CSNs with the capacity for precise temporal integration. We demonstrate that stimulus-specific CSNs act as critical relay sites, integrating convergent inputs to function as high-order coincidence detectors. Within a narrow temporal window, they combine precisely timed network signals, a process shaped by post-inhibitory facilitation driven by the precise temporal alignment of an upstream network activity that effectively maintains information between stimuli. Our network-centric view provides a mechanistic account for how auditory circuits bridge discrete sensory events to construct coherent, temporally extended percepts, consistent with experimental observations across species.

## Results

To evaluate how stimulus-specific CSNs achieve selective integration over extended delays, we began by examining the canonical mechanisms proposed for two-tone combination processing in the auditory system. These include: (1) temporal summation, in which successive subthreshold excitatory inputs combine to drive the neuron above firing threshold,^32^^,^^33^ (2) dual post-inhibitory rebound (PIR), in which two inhibitory inputs interact with the intrinsic properties of the CSN, particularly low-threshold T-type calcium channels, to produce a rebound burst in the CSN^3^^,^^34^^,^^35^; and (3) postsynaptic facilitation, the most widely recognized mechanism, in which an initial inhibitory input primes the CSN by de-inactivating calcium channels and establishing a transient window of heightened excitability that enables a subsequent excitatory input to trigger robust spiking.^3^^,^^13^^,^^15^^,^^18^^,^^26^^,^^36^^,^^37^ While each of these mechanisms can -account for short-timescale combination sensitivity, it remains unclear whether they are sufficient to explain the extended temporal integration observed in songbirds. We therefore developed computational models to test these scenarios systematically, and then extended them into a biologically realistic network framework incorporating upstream dynamics. This approach enabled us to identify the limits of simplified motifs and to uncover how network-level mechanisms generate the temporally precise, stimulus-pair sensitivity characteristic of songbird auditory circuits.

### Temporal limits of simple circuits in stimulus-pair integration

**Figure 1:**
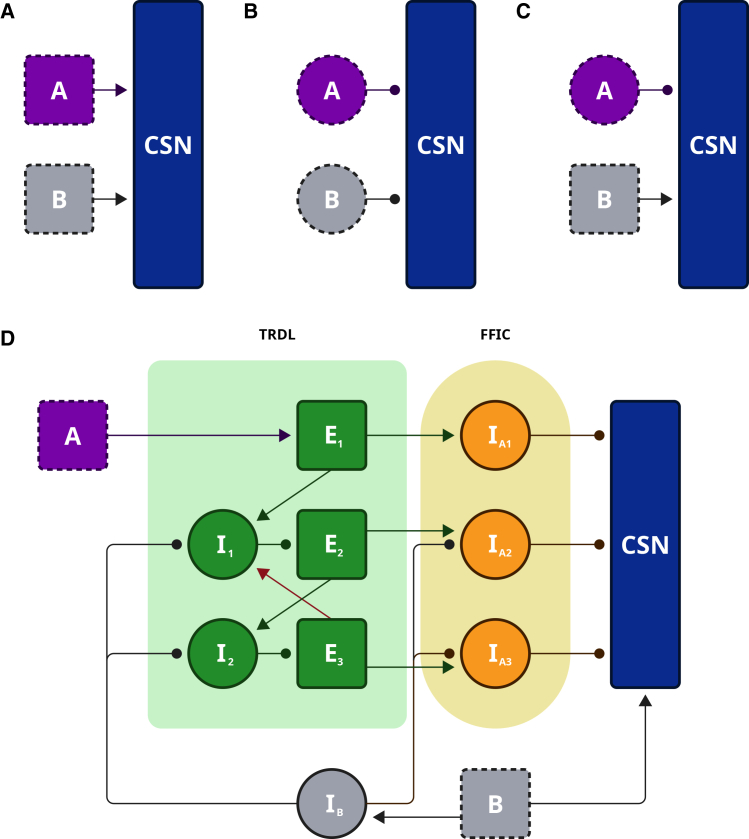
Network architectures for two-stimulus synaptic integration and combination sensitivity (A) Temporal summation model showing convergent excitatory inputs from neurons A (purple) and B (gray) onto the combination-sensitive neuron (CSN, navy). (B) Dual post-inhibitory rebound (PIR) model showing convergent inhibitory inputs from interneurons A and B onto the CSN. (C) Postsynaptic facilitation model showing convergence of an inhibitory input from interneuron A and an excitatory input from neuron B onto the CSN. (D) Layered network model for combination sensitivity. The circuit includes an A-selective excitatory neuron (dashed purple), a B-selective excitatory neuron (dashed gray), an inhibitory interneuron I_B_ (gray), a combination-sensitive neuron (CSN, navy), a transient reverberatory delay loop (TRDL, green), and a feedforward inhibitory convergence pathway (FFIC, yellow). The TRDL contains excitatory neurons E_1_-E_3_ and inhibitory interneurons I_1_-I_2_. The FFIC contains three interneuronal populations, I_A1_-I_A3_. Excitatory neurons are shown as squares and inhibitory neurons as circles; excitatory synapses are indicated by solid arrowheads and inhibitory synapses by rounded arrowheads. The CSN is represented by a rectangle.

We started by developing a testable network model for each of these three mechanisms, with the goal of evaluating the extent to which these would explain the observed neuronal behavior in songbirds, particularly for a stimulus-pair combination sensitivity. In all cases, we represent paired inputs as two abstract stimuli, denoted as A and B, which correspond to the functional activation of distinct, stimulus-selective upstream neurons (presynaptic to the CSN) rather than explicit acoustic syllables. Figures 1A–1C illustrates the network architectures for the different connectivity patterns considered above: a configuration in which the CSN receives two excitatory inputs selective for stimuli A and B (Figure 1A), a configuration with two inhibitory inputs selective for A and B (Figure 1B), and a mixed configuration in which the CSN receives an inhibitory input from stimulus A and an excitatory input from stimulus B (Figure 1C).

For each of the simple network scenarios shown in Figure 1, we simulated the underlying network dynamics under different conditions. Throughout this paper, we refer to the stimulus that activates the selective neuron A as the first stimulus (or S1), and to that activating the selective neuron B as the second stimulus (or S2). Starting with the temporal summation scenario (Figure 1A), the firing patterns of the neurons representing S1, S2, and CSN are shown in Figure S2, when both stimuli arrive sequentially (Figure S2A), when the firing of S2 is delayed by 50 ms from the offset of S1 (Figure S2B), when the temporal order of arriving stimuli is reversed (Figure S2C), and when the CSN receives the same excitatory stimuli twice (for example, when repeated S1, Figure S2D). When both stimuli arrive in succession, the first input elevates the CSN’s membrane potential to a subthreshold level, increasing its excitability and enabling the generation of a facilitative response triggered by the second input that arrives within a strict temporal window (Figure S2A). A short delay before the arrival of the second stimulus results in a combination failure for this scenario (Figure S2B), because even a delay as short as 50 ms is sufficient to bring neuron A’s membrane potential back to its resting state, rendering S2’s effect insufficient to trigger a suprathreshold response in the CSN. More importantly, this network seems to be insensitive to the temporal order of stimuli, as reversing their order yields successful behavior (Figure S2C). Stimulus-specific selectivity was also absent, since the CSN is stimulated by the summation of subthreshold inputs that share similar input characteristics (repeated S2, Figure S2D). We performed similar simulations for the second scenario (Figure 1B), where the CSN receives dual inhibitory inputs (Figure S3). The resulting network behavior closely resembles that observed in the first scenario. These findings render such simplified network structures incapable of explaining the mechanism of stimulus-pair combination sensitivity.

A substantial body of physiological work in songbird HVC strongly supports the need for a more structured circuit architecture than these simple motifs allow. Intracellular and juxtacellular recordings in zebra finch HVC demonstrate that inhibitory interneurons respond at remarkably short latency and with broad tuning to birdsong,^19^^,^^20^ whereas projection neurons (HVC_RA_ and HVC_X_) fire sparse, temporally precise bursts that are shaped by this early inhibition.^19^^,^^38^ Dual recordings in Rosen and Mooney^20^ further revealed strong synaptic interactions between interneurons and projection neurons, consistent with fast feedforward and feedback inhibition within a local microcircuit.^20^ Comparable dynamics are observed in upstream auditory/nidopallial regions, where GABAergic interneurons exert early, strong control over auditory responses,^39^ and where inputs from NIf engage temporally staggered excitatory and inhibitory pathways into HVC.^40^ These studies collectively support an architecture in which early, reliable inhibition and delayed excitation coexist, providing a realistic biological foundation for the structured delay-dependent motif we examine below.

**Figure 2:**
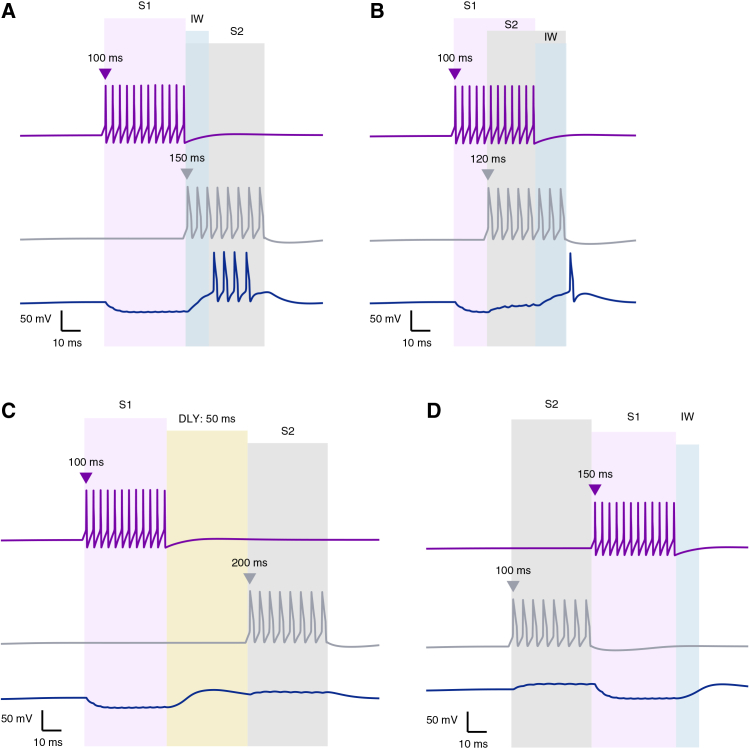
Simple postsynaptic facilitation supports only short-timescale stimulus association Representative voltage traces from the simplified postsynaptic facilitation model are shown for an inhibitory interneuron responding to the first stimulus (S1, purple), an excitatory neuron responding to the second stimulus (S2, gray), and the combination-sensitive neuron (CSN, navy) under different temporal arrangements of the two stimuli. Purple and gray triangles indicate stimulus onset, and shaded regions indicate stimulus duration. (A) S1 (50 ms) followed immediately by S2 (50 ms) produces successful facilitation at the CSN, generating a post-inhibitory rebound burst. The light blue shaded region denotes the integration window (IW), corresponding to a brief period of heightened CSN excitability. (B) When S2 arrives before the end of S1, facilitation fails. (C) When S2 is delayed by 50 ms beyond S1 offset, the delay interval (DLY; light yellow shaded region) exceeds the effective integration window and the CSN fails to generate an associative response. (D) Reversing stimulus order abolishes facilitation and produces dissociated responses. Traces are representative simulations under the indicated stimulus conditions. See also Figures S2 and S3.

Accordingly, in this work we focus on the postsynaptic facilitation (or priming) mechanism illustrated in Figure 1C, in which the CSN receives temporally offset inhibitory and excitatory inputs driven by distinct stimuli. This mechanism has been widely proposed as a circuit-level basis for syllable combination sensitivity in songbirds.^4^^,^^15^^,^^18^ To characterize its dynamics, we analyzed the response of the simplified network under four conditions: when the excitatory input coincides with the offset of S1 (Figure 2A), when the onset of S2 precedes the end of S1 (Figure 2B), when the arrival of S2 is delayed by 50 ms (Figure 2C), and when the temporal order of the two stimuli is reversed (Figure 2D).

In the case of successful integration (Figure 2A), interneuron A fires at the onset of S1 (t = 100 ms), delivering an inhibitory input to the CSN that persists for the duration of the stimulus (50 ms, shaded purple area). This hyperpolarization primes the CSN, which begins a rebound response at stimulus offset (t ∼150 ms) due to its T-type Ca^2+^ and H- currents (see STAR Methods). The resulting rebound window, lasting for a few milliseconds defines the CSN’s integration window (IW, shaded light blue), during which its intrinsic excitability is enhanced. When S2 activity overlaps with this window, the excitatory input coincides with the rebound depolarization, driving the CSN above threshold and producing a maximal facilitatory response that signifies successful stimulus combination.

Temporal variation in the arrival of the second stimulus disrupts effective integration at the CSN. When S2 arrives prematurely, before the offset of S1 (e.g., t = 120 ms, Figure 2B), or arrives outside the integration window, for instance, with a 50 ms delay (Figure 2C), the CSN fails to generate a combined response and instead exhibits two temporally dissociated subthreshold events. In the former case (Figure 2B), the excitatory effect of S2 counterbalances the inhibitory effect of S1, leading to a quasi-neutral effect that leaves the membrane potential of the CSN near resting levels. In the latter case (Figure 2C), the short delay after S1 offset (DLY of 50 ms, soft yellow shaded area) is not sufficient to prime the CSN to a level at which the excitatory effect of S2 can benefit from this priming and drive a response. Likewise, reversing the temporal order of the two stimuli abolishes coincidence detection, as the CSN fails to reach spiking threshold under this condition (Figure 2D).

*In vivo* recordings combined with perturbation experiments show that HVC activity during singing exhibits signatures of distributed recurrence and that local circuit interactions contribute directly to song timing.^41^ These observations support the biological plausibility of delay-generating mechanisms that extend strict feedforward processing and motivate our introduction of a transient reverberatory delay loop (TRDL) (detailed below) as a circuit substrate for extended temporal integration. Consistent with this view, converging evidence suggests that HVC and its afferent pathways engage recurrent or reverberant dynamics capable of sustaining activity beyond the duration of external input.^42^^,^^43^

One of the main limitations of simplified network models, is that they primarily focus on postsynaptic events taking place at the CSN level, which cannot fully explain behaviors that operate on larger timescales. We therefore propose that delay-dependent modulation is primarily implemented by upstream network interactions, presynaptic to the CSN, which dynamically shape the temporal window over which postsynaptic facilitation can occur.

### Layered network architecture for stimulus-pair association across extended timescales

To address the complexities of combination-sensitive behavior in songbirds, we expanded the simplified circuits into a layered network architecture. The expanded model incorporates a TRDL to provide a circuit-level substrate for temporal integration (Figure 1D) and a feedforward inhibitory convergence (FFIC) pathway to modulate the output stage. The TRDL is represented by a green ensemble and consists of three excitatory neurons (E_1_ to E_3_) and two inhibitory interneurons (I_1_ and I_2_). We will show below how the structured interactions between neurons within the TRDL sustain a brief pattern of reverberatory activity initiated by S1. Specifically, activity in the “purple-green” pair (A-E_1_) is recruited at the onset of S1, while the green pairs (E_1_-I_1_, I_1_-E_2_, E_2_-I_2_, and I_2_-E_3_) propagate this activity forward in time. An excitatory feedback connection from E_3_ to I_1_ (red feedback arrow) closes the loop and enables the network to preserve stimulus-related dynamics beyond the physical duration of S1, effectively serving as a temporal buffer.

The reverberatory outputs converge onto an FFIC hub, consisting of three intermediate interneurons (I_A1_-I_A3_) that translate the loop’s dynamics into a structured inhibitory drive. Conceptually, each interneuron in this hub can be considered an “interneuronal population”, mechanistically, we are representing each one as a single interneuron for the ease of applicability. These interneurons receive sequential excitatory input from E_1_, E_2_, and E_3_, respectively, and transmit temporally structured inhibition onto the CSN. Functionally, this sequence translates the reverberatory dynamics from the green delay loop into inhibitory signals that modulate the excitability of the CSN during the temporal delay period, hyperpolarizing its membrane potential.

In parallel, a gray-colored pair, representing an interneuron I_B_ (circle) and an excitatory neuron (B, dashed square) lies outside of these ensembles. The activation of this pair is driven by the onset of S2 and provides direct excitatory input to the CSN while also providing targeted inhibitory feedback to elements of both the green and yellow ensembles (I_B_-I_1_ and I_B_-I_2_ in the green ensemble, and I_B_-I_A2_ and I_B_-I_A3_ in the yellow ensemble). The disinhibitory function of this pair thus serves to regulate the decay of activity within the loop and ensuring that temporal integration remains transient and aligns with biologically realistic timescales, as will be described next. Eventually, this stops the inhibitory drive onto the CSN, enabling its post-inhibitory rebound to coincide with the and direct excitatory input from neuron B. In the following sections, we will explore how the interaction between these signals defines a narrow coincidence-detection window in which the CSN is selectively driven to spike only when S2 arrives within an appropriate temporal interval following S1.

Notably, although we illustrate groups of neurons using specific cell counts, these numbers are not meant to represent fixed anatomical quantities. Instead, they constitute a minimal circuit sufficient to express the mechanism. We assume that, under biological conditions, the same computation could be implemented by either larger or smaller neuronal ensembles. Thus, our formulation should be understood as a mechanistic abstraction rather than a literal cell count.

**Figure 3:**
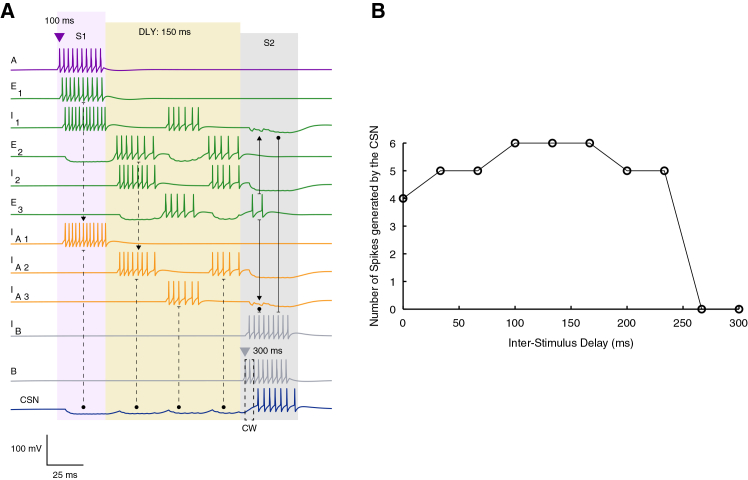
Delay-dependent network dynamics sustain combination-sensitive responses over a finite temporal range (A) Representative voltage traces from the full network model in response to S1 and S2 separated by a 150 ms delay (DLY, light yellow shaded region). Traces are color-coded according to the circuit schematic in Figure 1D, and neuron identities are indicated on the left. Dashed and solid arrows highlight key events in the temporal propagation of activity through the network. The coincidence window (CW) at the CSN is indicated by the dashed rectangle. Excitatory synaptic connections are denoted by solid arrowheads and inhibitory synaptic connections by rounded arrowheads. (B) CSN spike count as a function of inter-stimulus delay.

The activity patterns generated by the full model architecture during a 150-ms inter-stimulus delay are shown in Figure 3A. Although the same principles operate at shorter delays, the 150-ms condition makes it easier to visualize how the two key ensembles, that is the TRDL (green ensemble) and the feedforward inhibitory convergence pathway (yellow ensemble), jointly maintain temporally structured activity throughout the gap between stimuli. When the first stimulus (S1) is presented at 100 ms (purple triangle above neuron A’s firing trace), the A-selective excitatory neuron fires for 50 ms and initiates the delay loop by driving E_1_. Once activated, E_1_ propagates activity through two coupled inhibitory-excitatory pairs (E_1_→I_1_→E_2_ and E_2_→I_2_→E_3_). Because each inhibitory interneuron transiently hyperpolarizes its downstream excitatory partner, the offset of inhibition generates post-inhibitory rebound, producing a sequence of offset-triggered spikes in E_2_ and E_3_. Crucially, the firing of E_3_ feeds excitatory input back to I_1_, closing the loop. This recurrent offset-driven activity produces a reverberatory chain of rebound events that spans the entire 150-ms delay, effectively preserving temporal information about S1 after its offset. In parallel with initiating the loop, E_1_ also activates the first interneuron in the yellow FFIC pathway (I_A1_). This interneuron does not participate in the rebound sequence but instead provides the initial inhibitory input that hyperpolarizes the CSN (trajectory from E_1_→I_A1_→CSN traced by dashed lines).

As the delay loop progresses, each rebound spiking neuron in the green ensemble recruits the next interneuron in the yellow pathway: E_2_ activates I_A2_, and E_3_ activates I_A3_. This triggers a sequential transmission of inhibitory signals onto the CSN, leading to a structured hyperpolarization that persists throughout the entire delay (traced by dashed arrows) and that is shaped by the timing of rebound events within the loop (soft yellow shaded area). This inhibitory convergence is the key mechanism that primes the CSN (a concept that will be detailed in the following sections), building up intrinsic excitability (primarily by de-inactivation of T-type Ca^2+^ channels and activation of H-current) in preparation for the arrival of S2.

At t = 300 ms (reflecting 50 ms S1 duration + a 150 ms delay duration), presentation of the second stimulus (S2) activates the B-selective excitatory neuron. Neuron B plays two essential roles, first is the activation of I_B_, which suppresses activity in both the green (I_1_, I_2_) and yellow (I_A2_ - I_A3_) interneuronal populations, while the second role is to provide the postsynaptic drive required for coincidence detection through a direct excitatory input to the CSN. I_B_ therefore performs targeted disinhibition by shutting down the interneurons generating the inhibitory barrage. Under these conditions, the silencing of active interneurons is mediated through competing inhibitory-excitatory synapses, as shown by the lined arrows in the figure. This releases the CSN from inhibition at the exact time when the excitatory input arrives. Because the CSN has been primed during the delay, this release produces a strong rebound depolarization that coincides with the incoming excitatory drive from neuron B. The coupling of these two depolarizing influences, that is the intrinsic rebound and synaptic excitation, creates a tight coincidence detection window (CW), allowing the CSN to fire maximally when S2 arrives within the appropriate temporal interval.

Together, these dynamics illustrate how the TRDL maintains a temporally structured representation of S1 across extended delays, while the feedforward inhibitory convergence pathway controls CSN hyperpolarization and prepares its intrinsic excitability state. Upon arrival of S2, a precisely timed disinhibition-excitation sequence allows the CSN to act as a coincidence detector, generating combination-sensitive facilitation only for appropriate A-B timing.

### Dynamics of delay-dependent priming: Shifting from integration to coincidence detection

**Figure 4:**
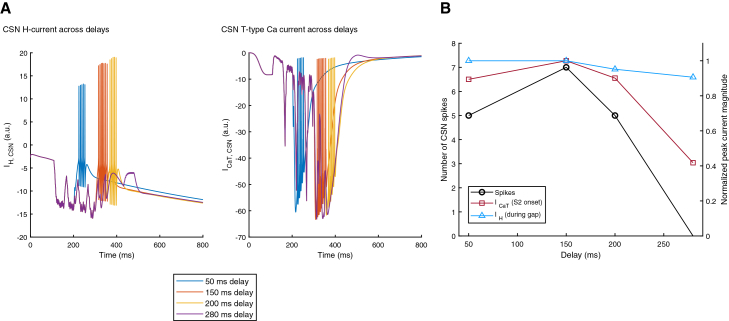
Delay-dependent intrinsic priming in the CSN (A) Representative time courses of the H-current ($I_{H}$, left) and T-type calcium current ($I_{CaT}$, right) in the CSN at inter-stimulus delays of 50, 150, 200, and 280 ms. Both currents show strongest rebound-related recruitment at intermediate delays (150–200 ms), followed by reduced activation at longer delays (280 ms), indicating that effective postsynaptic facilitation depends on the temporal co-engagement of these intrinsic currents. (B) CSN spike count (black circles) and intrinsic current amplitudes as a function of inter-stimulus delay. CSN spiking increases from short delays to a maximum at 150 ms and then declines at longer delays. A similar delay dependence is observed for the peak T-type calcium current at S2 onset (red squares) and the H-current during the delay interval (blue triangles), both of which increase up to approximately 150 ms and decrease thereafter.

At the network level, the coordinated interaction between the green TRDL and the yellow FFIC generates a structured shift between two temporal coding regimes. Following S1 onset, offset, and throughout the delay duration (150 ms in the above scenario), the network operates in a temporal integration mode. During this phase, the sequential activity of the I_A1_→CSN, I_A2_→CSN, and I_A3_→CSN inhibitory streams maintains the CSN in a hyperpolarized state, allowing it to encode the duration of inhibition. As this sustained inhibition progresses, intrinsic gating variables, particularly the de-inactivation of T-type Ca^2+^ channels and the activation of the H-current, gradually accumulate (Figure 4). In biophysical terms, when the CSN membrane is hyperpolarized, a gradual negative (inward) increase in $I_{H}$ is seen across delays (Figure 4A), see the relevant STAR Methods section for additional information. Upward current deflection refers to the time when the neuron returns toward rest or depolarization (that is, when the inhibitory phase stops), showing a decay in $I_{H}$ over milliseconds. Thus, the largest $I_{H}$ transients, in this case at the 150 ms delay condition, refer to the strongest post-inhibitory depolarization and are often associated with more spikes (Figure 4B).

Importantly, in our model the CSN operates within a physiologically realistic voltage range during the delay, typically between ∼–70 and −90 mV. In this voltage domain $I_{H}$ is partially activated, and begins to rise steeply at potentials that are more negative than ∼–90 to −100 mV. Likewise, when the membrane repolarizes above ∼–70 mV, $I_{H}$ rapidly deactivates. Thus, in our simulations $I_{H}$ is recruited over a moderate hyperpolarization range that is sufficient to contribute meaningfully to rebound depolarization without requiring extreme hyperpolarization (e.g., −110 to −120 mV). This makes our model consistent with HVC physiology,^44^^,^^45^ where sag and rebound are observed without the membrane ever entering deep hyperpolarized states.

An important distinction for the T-type calcium channel is that channels do not open at hyperpolarized potentials, but are rather de-inactivated (during hyperpolarization, $r_{T}$, which is the dynamic gating variable controlling de-inactivation “availability” moves to values that allow them to open later). During rebound/depolarization (that is at the onset of S2), the T-type channels activate, causing inward Ca^2+^ current that can push the neuron’s membrane over threshold and generate a Ca^2+^ burst, leading to higher and more sustainable intracellular elevations (see the calcium trace). Thus, the T-type current traces in Figure 4A show when and how strongly the CSN recruits T-type Ca^2+^ current for rebound at each delay. In essence, when S2 arrives at delay durations between 150 ms and 200 ms and depolarizes the CSN, there are stronger and more coherent T-type current transients than those seen at, say, 50 ms delay. Interestingly, at a 280 ms delay, much of the gating reset and hyperpolarization from S1 has decayed. As S1 ends and neuron B starts firing, it sees a more “naive” cell, or a less pre-de-inactivated $I_{CaT}$, and the rebound becomes weaker. This implies that larger and more temporally aligned T-type currents, the CSN exhibits a stronger rebound and generates more spikes (Figure 4B).

Thus overall, as hyperpolarization activates H-currents and removes inactivation from low-threshold Ca^2+^ channels, it makes the CSN more capable of producing a rebound depolarization once the inhibitory drive stops (at the end of the delay period). This dynamic change of state in the CSN, making it more excitable and “ready to fire” relative the delay duration is what we refer to as “delay-dependent priming.” This is reflected in our model, whereby Figure 4B shows that as the temporal gap between S1 offset and S2 onset increases from 0 to ∼150 ms, both $I_{CaT}$ availability and $I_{H}$ activation rise (for more details on quantification of intrinsic currents across delays, refer to the relevant section in the STAR Methods), resulting in an increase in the number of spikes generated by the CSN once S2 arrives. For delays exceeding ∼200 ms, these intrinsic currents begin to decay, the priming state collapses, and CSN firing correspondingly starts diminishing beyond ∼230 ms.

To quantify the temporal limits of this integration process, we systematically varied the inter-stimulus delay from 0 to 300 ms in ∼20-ms steps and measured CSN spike counts (Figure 3B). The model exhibited a clear delay-dependent profile: CSN output increased modestly from 4 to 5 spikes between 0 and ∼50 ms, remained stable between ∼50 and ∼100 ms, and was followed by a further increase to 6 spikes as delays reached between ∼100 and 150 ms. After that, spike counts showed a gradual decrease, particularly for delay durations between ∼150 and 200 ms as the number of spikes reverted to 5 before showing a sharp decline after ∼230 ms. At ∼260 ms delay, combination behavior is lost, as reflected by zero spikes.

Results from Figure 3 thus show that, once S2 arrives (at its onset), provided that the delay loop has propagated correctly, the network transitions into a coincidence detection mode. This coding regime is brief, lasting only a few milliseconds, and emerges from the convergence of two factors: (1) the CSN’s intrinsically generated rebound depolarization following release from inhibition, and (2) the precisely timed excitatory input arriving from the B-selective neuron. It is only when these depolarizing events coincide that the CSN generates its characteristic facilitated burst, thereby establishing the temporal specificity required for combination-sensitive responses.

In the sections that follow, we extend the analysis of this interaction, focusing in particular on how combined variations in delay duration and intrinsic conductances (T-type Ca^2+^ and H-current) shape CSN spiking output. This exploration quantitatively supports the delay-dependent priming mechanism described previously and clarifies the distinct contributions of each intrinsic current to the CSN’s response profile. We also map the range of synaptic conductance values that permit successful facilitation, thereby identifying the synaptic constraints under which the circuit reliably generates combination-sensitive behavior.

### Sensitivity to temporal order and the loss of facilitation

Before proceeding, it is important to illustrate the all-or-none, selective nature of these CSNs, which respond robustly only when the stimulus pair is presented in the correct temporal order. As illustrated previously, our model emphasizes that the timing of stimuli onsets and offsets, and their ordering, governs the transition from the temporal integration window to the brief coincidence detection window. This ordering is essential, as only the correct S1→S2 sequence drives the network into the appropriate primed state and subsequently enables coincidence detection at the CSN.

**Figure 5:**
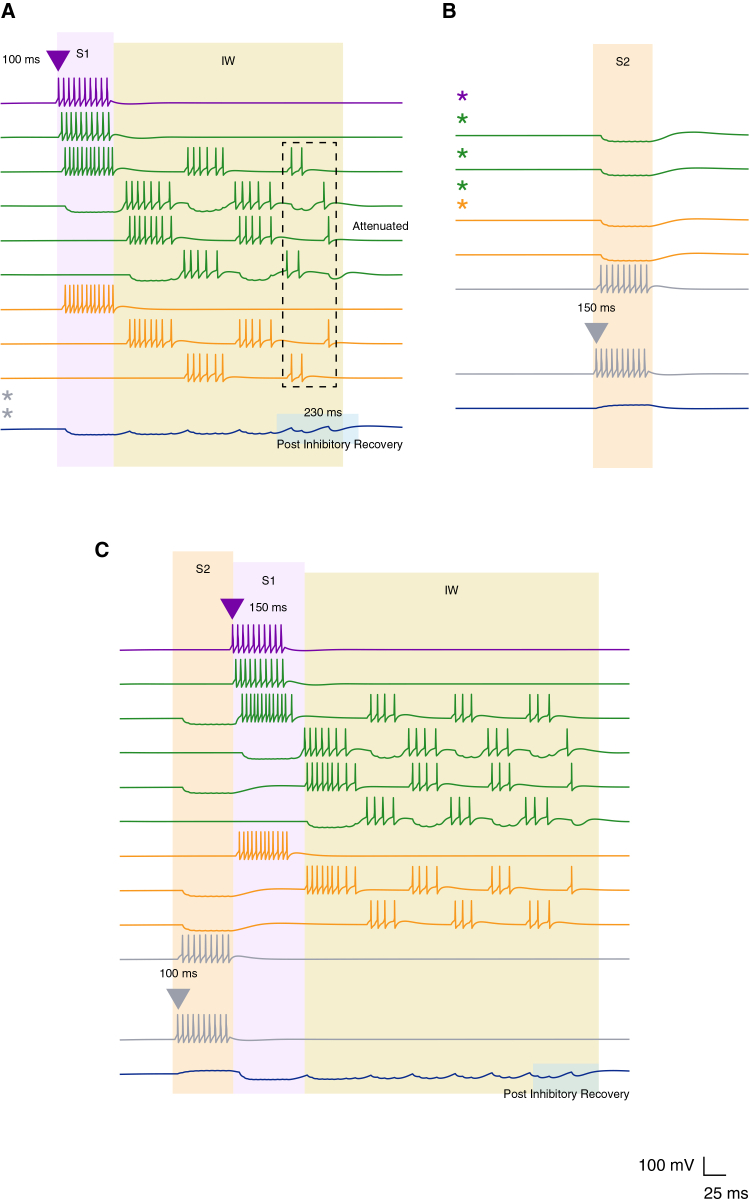
Network responses under conditions that fail to produce combination-sensitive output (A) Representative voltage traces showing the network response to presentation of S1 alone. S1 onset (purple triangle) initiates a cascade of firing (light purple shaded region), and S1 offset defines the integration windo (IW, light yellow shaded region). As the IW progresses, activity within the transient reverberatory delay loop gradually attenuates (dashed rectangle), weakening the inhibitory drive to the CSN and leading to a gradual post-inhibitory recovery (light blue shaded region around the CSN trace) rather than a facilitated response. (B) Representative voltage traces showing the response to presentation of S2 alone (gray triangle indicates S2 onset). (C) Representative voltage traces showing the response to reversed stimulus order, with S2 preceding S1 at 0 ms delay. Under this condition, the CSN shows gradual post-inhibitory recovery toward the end of the IW rather than the sharp release required for robust spiking. Voltage traces from neurons not activated under a given condition are omitted and replaced by color-coded asterisks.

To further illustrate this principle, we examined how disrupting the S1→S2 sequence, either by presenting only a single stimulus or by reversing the stimuli’s order, leads to a failure to generate the appropriate combination-sensitive response (Figure 5). These conditions reveal that the mechanisms described previously do not generalize indiscriminately; rather, the network requires the precise temporal structure of the “expected stimulus pair” for successful facilitation.

When only the first stimulus (S1) is presented (Figure 5A), its onset at t = 100 ms initiates activity in the upstream circuit that opens the temporal integration window, which extends for approximately 260 ms (soft yellow shading). During this interval, the patterned interactions between the TRDL (green ensemble) and the feedforward inhibitory convergence pathway (yellow ensemble) maintain inhibition onto the CSN. However, this inhibitory drive weakens progressively as the rebound activity within the green excitatory neurons diminishes across successive cycles of hyperpolarization (dashed rectangle). As firing in the yellow interneuron population also decreases, CSN hyperpolarization correspondingly lessens (see intrinsic explanation above and in Figure 4), allowing the membrane potential to drift back toward baseline without generating a robust rebound depolarization (light blue shading). This implies that the CSN can no longer support a rebound-mediated coincidence response once this temporal limit has passed, as is the case when a second stimulus never arrives. Therefore, although the offset of S1 initiates temporal integration, S1 alone is insufficient to elicit CSN spiking, highlighting the role of the CSN as a coincidence detector.^3^^,^^5^^,^^13^^,^^15^^,^^27^^,^^46^^,^^47^

Similarly, S2 presented in isolation fails to evoke CSN firing (Figure 5B). Its excitatory input alone produces only subthreshold depolarization in the CSN, and without upstream activity the disinhibitory role of IB is effectively lost. Reversing the temporal order of the stimuli (S2→S1) likewise abolishes combination-sensitive responses (Figure 5C). This reversed sequence produces dissociated activity patterns similar to those observed in the simplified model (Figure 2C): S2 alone provides insufficient priming, and the delayed arrival of S1 fails to engage coincidence detection. These outcomes the temporal trajectory required along the network for effective stimulus association, supporting an order-dependent transition between temporal coding regimes: from temporal integration triggered by the offset of S1 to coincidence detection triggered by the onset of S2.

To confirm that the observed postsynaptic facilitation is not attributable to simple within-stimulus linear temporal summation, we examined repeated presentations of the same stimulus. Both S1-S1 (Figure S4A) and S2-S2 (Figure S4B) produced responses equivalent to isolated S1 or S2 presentations, demonstrating that our network, indeed, does not respond through nonspecific temporal summation but instead operates within stimulus-specific selectivity. Together, these findings highlight that temporal combination sensitivity across extended timescales emerges from the coordinated interaction of circuit-level dynamics, including excitation-inhibition interplay and precisely timed temporal windows, rather than from simple neuron-level mechanisms.

### Synergistic contributions of $g_{CaT}$ and $g_{H}$ to CSN rebound excitability

We started our analyses of interaction by examining the role of intrinsic membrane conductances in shaping CSN responses. We focused on the two key intrinsic currents, the H-current and the T-type calcium current, and quantified how changes in their conductances influence CSN spiking. In the default simulation used to generate the network dynamics described previously, the maximal H-current conductance ($g_{H}$) was set to 6 nS and the maximal T-type calcium conductance ($g_{CaT}$) to 0.4 nS. For this analysis, we simulated the network at a 50 ms inter-stimulus delay rather than 150 ms, as the two conditions produced qualitatively similar behavior while the shorter delay provided a simpler illustration.

**Figure 6:**
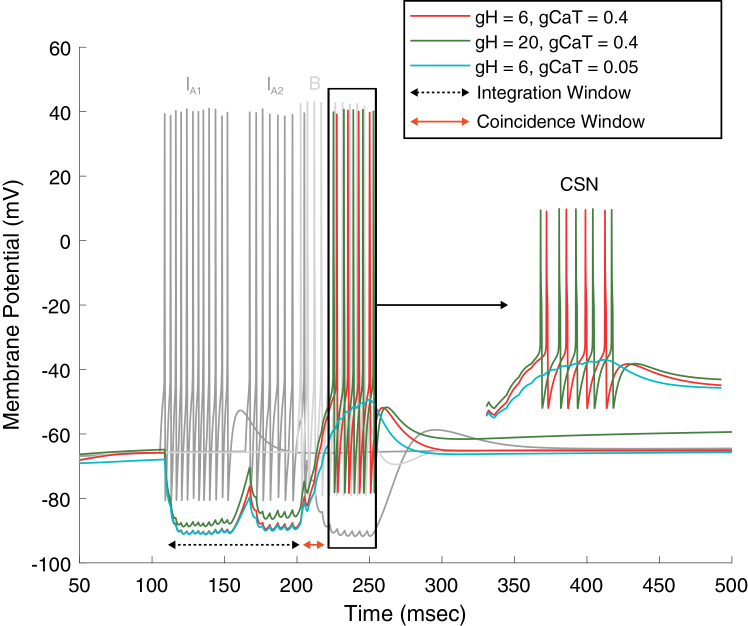
Intrinsic conductance changes alter CSN priming and rebound output Representative voltage traces at a 50 ms inter-stimulus delay showing the membrane potentials of interneurons I_A1_ and I_A2_ (dark gray), which provide inhibitory input to the combination-sensitive neuron (CSN), and neuron B (light gray), which provides the direct excitatory input to the CSN. Three overlaid CSN traces illustrate different intrinsic conductance conditions. The firing of I_A1_ and I_A2_ defines the integration window (black dashed line beneath the traces; 100 ms in this condition), followed by a brief coincidence window (solid orange line) associated with the onset of neuron B activity. The red CSN trace shows the default condition and produces a 4-spike rebound burst. The green trace shows increased $g_{H}$ with $g_{CaT}$ held constant, resulting in a larger 6-spike burst and reduced hyperpolarization during the integration window. The blue trace shows reduced $g_{CaT}$ with $g_{H}$ held at its default value, abolishing suprathreshold post-inhibitory rebound. The inset shows an expanded view of the rebound region outlined by the black rectangle.

At an inter-stimulus delay of 50 ms, the CSN generated 4 spikes (represented by the red voltage trace in Figure 6). The figure also represents voltage traces of I_A1_ and I_A2_ (dark gray traces) highlighting two features: first, the hyperpolarization of the CSN that occurs due to these two inhibitory inputs, and second, the duration of the integration window (black dashed line beneath the traces, reflected by the amount of hyperpolarization from I_A1_ and I_A2_). We also show the voltage trace of neuron B (lighter gray trace), which provides the direct excitatory input to the CSN and terminates the inhibitory transmission (both I_A1_ and I_A2_ stopped firing once B neuron started firing). Beneath the firing trace of B, a solid orange line marks the associated coincidence window.

We carried out two variations. First, we examined the effect of strengthening the hyperpolarization-activated current. Increasing $g_{H}$ from 6 to 20 nS while keeping $g_{CaT}$ fixed at 0.4 nS elevated CSN output from four to five spikes (green trace). With stronger H-conductance, the CSN depolarizes more rapidly following release from inhibition, and its membrane potential remains slightly more depolarized during the integration window (minimum ∼ -85 mV compared to ∼ -89 mV at baseline). This shift reflects an increased contribution of $I_{H}$, which accelerates the rebound trajectory and enhances the efficacy of the coincidence between rebound depolarization and B-input.

We then examined the case of reducing $g_{CaT}$, which profoundly abolished CSN spiking. When $g_{CaT}$ was decreased from 0.4 to 0.05 nS while $g_{H}$ remained at 6 nS, the CSN failed to produce a rebound spike altogether (blue trace). Despite adequate H-current activation and sufficient hyperpolarization to deinactivate T-channels, the greatly reduced T-type conductance provided insufficient inward Ca^2+^ current to generate a rebound burst. This demonstrates that while $I_{H}$ modulates excitability and contributes to rebound shaping, T-type calcium conductance is crucial for generating suprathreshold rebound responses.

Thus, these results highlight the intrinsic coordination within the CSN itself, whereby its spiking is conditioned upon a finely tuned interplay between two key intrinsic currents: H-currents bias the membrane toward rebound readiness, but T-type currents ultimately determine whether the rebound crosses threshold and produces a burst. Reversing stimulus order disrupted this synaptic-intrinsic interplay, preventing successful integration (Figure S5), consistent with postsynaptic priming models.^18^

**Figure 7:**
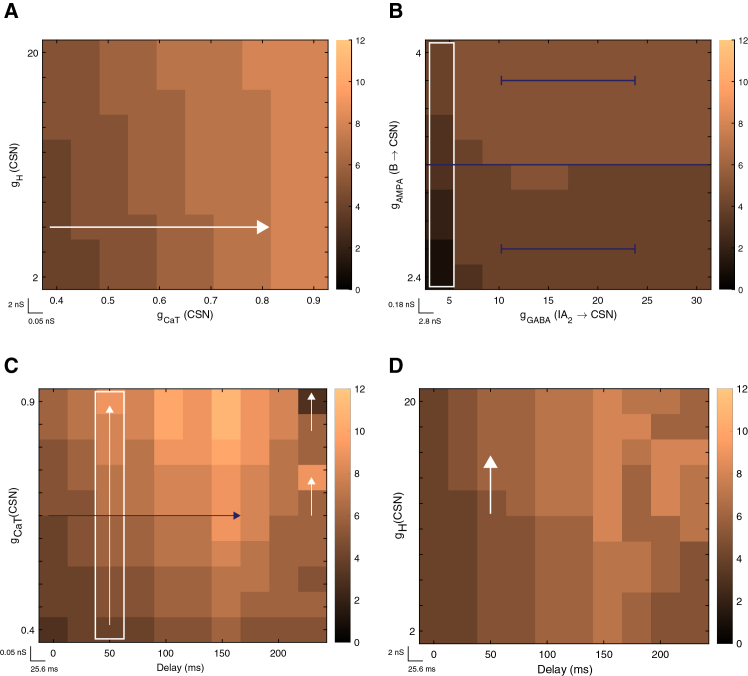
Intrinsic, synaptic, and delay-dependent determinants of CSN spiking (A) Heatmap showing CSN spike count as a function of intrinsic T-type calcium conductance ($g_{CaT}$; 0.4–0.9 nS in 0.05 nS increments) and H-current conductance ($g_{H}$; 2–20 nS in 2 nS increments), with all other parameters held at default values. A total of 96 simulations (12 × 8) were performed for this representation. (B) Heatmap showing CSN spike count as a function of inhibitory synaptic conductance ($g_{GABA}$, I_A2_⟶ CSN; 5–30 nS in 2.8 nS increments) and excitatory synaptic conductance ($g_{AMPA}$, B ⟶ CSN; 2.4–4 nS in 0.18 nS increments), with all other parameters held at default values. A total of 120 simulations (10 × 12) were performed for this representation. (C) Heatmap showing CSN spike count as a function of inter-stimulus delay (0–230 ms) and intrinsic $g_{CaT}$ conductance (0.4–0.9 nS). A total of 160 simulations (16 × 10) were performed for this representation. (D) Heatmap showing CSN spike count as a function of inter-stimulus delay (0–230 ms) and intrinsic $g_{H}$ conductance (2–20 nS). A total of 160 simulations (16 × 10) were performed for this representation. In all representations, each pixel represents one simulation under a unique parameter combination, and color indicates the number of spikes generated by the CSN according to the unified color bar.

To further explore the cooperative roles of $g_{CaT}$ and $g_{H}$ in gating the integration process, we systematically varied their conductances (Figure 7A). The intrinsic conductance interaction map shows a clean and correlated pattern that illustrates how $g_{CaT}$ and $g_{H}$ cooperate in shaping the excitability of the CSN. There appear to be strong horizontal gradient, whereby increasing $g_{CaT}$ consistently produces more spikes, with little dependence on the specific $g_{H}$ value. For example, at a fixed $g_{H}$ of 6 nS (where the white arrow starts), the CSN generates approximately 4 spikes, with a $g_{CaT}$ = 0.4 nS. Moving along the *x* axis, increasing $g_{CaT}$ to 0.5 nS yields ∼5 spikes, to 0.6 nS yields ∼6 spikes, to 0.7 nS yields ∼7 spikes, and 0.8 nS yields ∼8 spikes. This seemingly monotonic progression highlights the crucial and dominating role of $I_{CaT}$ availability in determining the strength of the rebound burst. Thus, the more T-type channels are available to carry inward current after release from inhibition, the more the CSN recruits spikes within the coincidence window.

Moving along the vertical axis, in contrast, that is, increasing $g_{H}$ values while keeping those of $g_{CaT}$ fixed, showed only slight variations in spike count, as evidenced by the nearly uniform color. This implies that although H-current contributes to setting the hyperpolarized state during the integration window, its effect on the final spike output is modulatory rather than generative. That is, while $g_{H}$ tunes the membrane state during inhibition, thereby enabling later spiking extension, $g_{CaT}$ scales the magnitude of the burst, acting as the primary driver of rebound spike generation. This cooperative interaction aligns well with the biophysical synergy described previously, underlying delay-dependent priming.

### Synaptic and intrinsic determinants of the integration-to-coincidence transition

We next examined how synaptic conductances shape CSN activity by assessing the sensitivity of its combination-sensitive response to changes in the strength of key synaptic inputs. Focusing on the inhibitory pathway from IA and the excitatory input from the B-selective neuron, we systematically varied the corresponding synaptic conductances ($g_{GABA}$ and $g_{AMPA}$, see STAR Methods) and quantified the resulting CSN spike output. Since we’ve performed this set of simulations at a 50-ms inter-stimulus delay, the relevant inhibitory input to the CSN at this timescale arises from I_A2_; accordingly, we examined the I_A2_→CSN and B→CSN connections.

Interestingly, varying either conductances generated little change in CSN spike counts (Figure 7B). It is only along the first column (enclosed by the white rectangle) that a noticeable change is encountered. This column refers to the least inhibitory conductance value yielding between 1 and 2 spikes when combined with low excitatory conductance values ranging from 2.4 nS to 2.9 nS. For higher values of AMPA conductance (between 3.6 nS and 4 nS), this 5 nS of GABAergic conductance generated 4 spikes. Aside from this observation, the heatmap seems to be made up of two blocks, divided at an AMPA conductance value of ∼3.2 nS (blue line). In each of these two blocks (below and above the blue line), and across the entire range of GABA conductance values, the CSN generated the same number of spikes, indicating that our network behavior is robust to moderate scaling of these synaptic weights. This indicates that while appropriate synaptic inputs are necessary to initiate the process, the qualitative behavior of the network—that is, its ability to generate a combination-sensitive behavior—is primarily governed by the intrinsic and temporal parameters of the circuits.

So far, our results support the core hypothesis that the network operates through a sequence of precisely coordinated temporal events. In particular, the offset-driven responses produced by the green neurons within the TRDL sustain reverberatory activity that preserves information about S1 throughout the delay period. This activity ensures that the system remains in a temporal integration mode until S2 arrives. The subsequent, tightly timed transition into a coincidence detection regime, marked by the time at which the CSN’s rebound depolarization converges with excitatory input from S2, is what enables reliable postsynaptic facilitation. Together, these layered interactions across synaptic inputs, inhibitory gating, and intrinsic membrane currents give rise to combination-sensitive responses as an emergent property of the entire circuit.

This unidirectional, dual-coding capacity, however, also raises the possibility that disruptions in either window can compromise the behavior. To examine the relationship between the temporal integration window (defined by inter-stimulus delay) and the coincidence detection window (defined by $g_{CaT}$ and $g_{H}$), we generated two-parameter interaction maps (Figures 7C and 7D).

For the delay—$g_{CaT}$ analysis (Figure 7C), the heatmap shows two prominent gradients in CSN spike output. The first is along the vertical axis, that is, by holding a fixed delay value (e.g., 50 ms, white rectangle) and moving upwards (increasing $g_{CaT}$ values), the number of CSN spikes gradually increases, as reflected by the progressive lightening of the color map. This aligns with the expected behavior while also reinforcing earlier results; that is, for a given delay, one that is long enough to hyperpolarize the CSN and de-inactivate T-type channels, larger $g_{CaT}$ values enable stronger T-current influx during rebound, which strengthen rebound depolarization and generates additional spikes.

A deviation from this gradient appears at the longest delay duration (230 ms, rightmost column), where spike output becomes less uniform. Notably, raising $g_{CaT}$ from 0.6 to 0.7 nS increases the CSN response from 6 to 9 spikes, whereas a further increase in $g_{CaT}$ to 0.9 nS resulted in a reduction to 3 spikes (white upward arrows). This reduction highlights the critical temporal alignment constraint, that is: at extended delays, the inhibitory drive onto the CSN begins to wane, allowing the membrane potential to relax back toward baseline (Figure 5A). Under these conditions, a high $g_{CaT}$ can trigger a weak, premature rebound that fails to align with the arrival of S2 excitation. In these cases, the few recorded spikes reflect independent excitation from neuron B rather than a true associative response (see Figure S6 for the underlying trace dynamics). Along the horizontal axis, a complementary dependency is observed. For a fixed $g_{CaT}$ value (purple arrow), increasing the inter-stimulus delay initially leads to a graded increase in CSN spike output, as indicated by the progressive lightening of the heatmap. This reflects the growing efficacy of rebound mechanisms as longer delays permit deeper and more prolonged hyperpolarization, thereby facilitating T-type channel deinactivation. However, this trend reaches a clear turning point at delays beyond 200 ms, where CSN spiking begins to decline, as evidenced by the re-darkening of the color map. Similar to moving along the vertical axis, this shows that CSN’s spiking response is not only controlled by the interaction between $g_{CaT}$ and the delay duration, but is also under the constraint imposed by temporal alignment. This loss of temporal overlap provides a mechanistic explanation for the decay of combination-sensitive behavior observed at extended delays. Consistent with this view, we previously showed that CSN responses weaken beyond ∼230 ms and are diminished at ∼260 ms (Figure 3B). Thus, effective combination sensitivity emerges within a finite temporal window in which intrinsic rebound dynamics and delayed excitation are appropriately aligned; as this window is exceeded, the system transitions from associative to independent processing of the two stimuli.

The delay - $g_{H}$ analysis (Figure 7D) revealed a broadly complementary pattern. Consistent with earlier findings, increasing $g_{H}$ produces a modest but consistent enhancement in CSN output up to delay of approximately 150 ms. Specifically, for a fixed delay, moving upward along the heatmap (increasing $g_{H}$) results in a slight lightening of the color map, indicating a small increase in spike count. For example, for a 50 ms delay, $g_{H}$ values between 2 and 12 nS consistently generated 5 spikes, whereas increasing $g_{H}$ to 14 nS and above, leads to a sixth spike (white arrow). This gradual effect is consistent with the role of $I_{H}$ in modulating membrane excitability and rebound amplitude, rather than directly generating spikes (Figures 6 and 7A). At longer delays (≥200 ms), the pattern becomes less uniform, which aligns with findings from the delay - $g_{CaT}$ analysis. Along the *x* axis, a similar delay-dependent structure is evident. For a fixed $g_{H}$ value, increasing the inter-stimulus delay leads to a gradual and modest increase in CSN spike output up to a cutoff around ∼150 ms. However, beyond this point, the heatmap darkens, indicating a reduction in spike count at longer delays as the state collapses. These maps collectively demonstrate that effective combination sensitivity emerges within a finite zone where intrinsic rebound dynamics and delayed excitation are precisely aligned.

Finally, we performed further analyses to assess the robustness of our network behavior in response to variations in stimulus characteristics, specifically stimulus duration and intensity. While preceding simulations used default values (50 ms duration, 200 pA intensity), we expanded the parameter space to include stimulus durations up to 200 ms and intensities up to 300 pA.

### Stimulus duration: Thresholds and saturation

**Figure 8:**
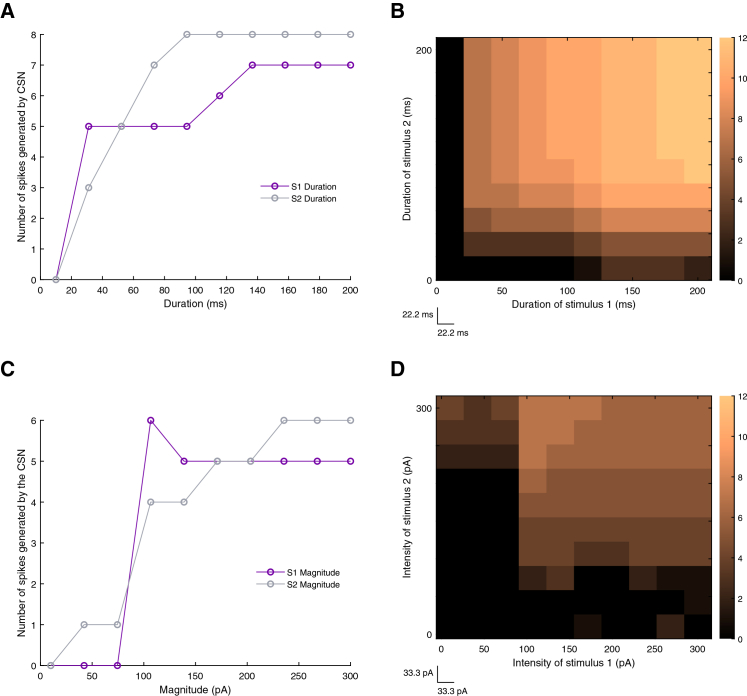
Stimulus duration and intensity shape CSN combination-sensitive output (A) Line plots showing CSN spike count as a function of S1 duration (purple) or S2 duration (gray), with the other stimulus held at its default value. Stimulus duration was varied from 0 to 200 ms. (B) Heatmap showing CSN spike count as a function of the duration of S1 and S2. Stimulus durations ranged from 10 to 200 ms in 22.2 ms increments. A total of 100 simulations (10 × 10) were performed for this representation. (C) Line plots showing CSN spike count as a function of S1 intensity (purple) or S2 intensity (gray), with the other stimulus held at its default value. Stimulus intensity was varied from 0 to 300 pA. (D) Heatmap showing CSN spike count as a function of the intensity of S1 and S2. Stimulus intensities ranged from 10 to 300 pA in 33.3 pA increments. A total of 100 deterministic simulations (10 × 10) were performed for this representation. In the heatmaps, each pixel represents one simulation under a unique stimulus-parameter combination, and color indicates the number of spikes generated by the CSN according to the unified color bar.

We first examined stimulus duration using a single-parameter variation approach, systematically varying the duration of one stimulus while holding the other constant. With an interstimulus delay fixed at 50 ms, we explored durations from 10 to 200 ms for each input. As shown in Figure 8A, very brief presentations of the first stimulus (∼10–20 ms, purple line) were insufficient to initiate CSN firing. However, once the duration crossed a threshold (∼30–40 ms), the CSN began to fire reliably, generating 5 spikes, and plateaued at approximately 100 ms. Another minor increase in the spike count can be seen for S1 duration values between 100 and 140 ms, reaching a maximal value of 7, which then also plateaued again beyond 140 ms. For S2 duration values (gray line), similar to S1, very short durations (∼10–20 ms) were insufficient to trigger spiking. However, once S2 duration increased to ∼100 ms (exceeding ∼30 ms), CSN spike count grew more steeply than for S1. Once S2 was long enough (on the order of ∼100–150 ms), the curve also saturated and further increases in duration did not increase spike count.

Thus, while both stimuli need to have a minimal duration to trigger spiking, S2 duration has a stronger impact on the magnitude of the CSN response than S1 duration does. This pattern fits nicely with the mechanistic role of S1 and S2 in the model. S1’s job is to engage the delay loop and feedforward inhibitory convergence, and to keep the CSN hyperpolarized long enough to prime the CSN, putting it in a highly excitable state, until S2 arrives, which explains why short durations fail to evoke spiking. Once the priming state saturates, the CSN is already “ready” so a longer S1 duration does not translate to more spikes. On the other hand, S2’s role is to provide the main excitatory drive that reads out the primed state and determines the magnitude of the facilitated burst. Consequently, once intrinsic burst mechanisms reach their saturation point, further increases in S2 duration no longer amplify the response.

To generalize these findings, we co-varied the durations of both stimuli across the full 10 to 200 ms range while maintaining default conductance parameters and a 50 ms interstimulus delay. The resulting heatmap (Figure 8B) confirms the line-plot results. When either stimulus is too brief, the upstream circuitry cannot generate sufficient inhibition to prime the CSN, nor can S2 provide enough excitation to elicit a rebound burst. The distinct mechanistic roles of the two stimuli are reflected directly in the heatmap structure: moving along the *x* axis (increasing S1 duration) produces progressive brightening, especially when paired with longer S2 durations, whereas moving along the *y* axis (increasing S2 duration) produces most of its changes below ∼100 ms, after which the map becomes more uniform.

Together, these analyses highlight two important findings. First, combination-sensitive responses emerge only when both stimuli exceed their minimal temporal requirements, and second, S2 duration is an important determinant of the magnitude of postsynaptic facilitation.

### Stimulus intensity: Asymmetric contributions

For stimulus intensity, we applied the same single-parameter variation strategy, systematically altering the intensity of one stimulus over a defined range while keeping the other fixed at its default value (Figure 8C). When either S1 (purple line) or S2 (gray line) is below ∼100 pA, the CSN does not fire, as both curves appear to sit near 0–1 spikes in that low-intensity range. As S1 intensity increases beyond ∼100 pA, the number of spikes rises abruptly to 6 and then stabilizes around 5 as intensity increases further toward 300 pA. For S2, the increase in intensity had a broader dynamic range, whereby raising S2 from ∼100→150→ 200→250→300 pA gradually pushes the CSN from ∼4→5→6 spikes. This again confirms the role of S1, acting as a “priming-driver”, whereas S2 provides the decisive depolarizing drive that coincides with the rebound. The flattening of both curves at a certain limit also reinforces that the CSN can only fire a finite burst within a narrow coincidence window; once the cell is driven into maximal rebound firing, the limit is set by intrinsic spike dynamics and not by input amplitude.

The two-dimensional intensity analysis (Figure 8D) further reinforced this functional asymmetry. Both stimuli exhibited a minimum intensity threshold for effective combination, as indicated by the absence of CSN spiking in the darkest regions of the plot. Increasing S1 intensity (*x* axis) yielded little to no change in spike count, whereas increasing S2 intensity (*y* axis) led to a clear progression from darker to lighter shades, reflecting higher spike counts.

Together, these stimulus-related analyses demonstrate that our model is not strictly dependent on rigid parameters but is instead robust to variations in input duration and strength. This flexibility allows the network to generalize across a range of input characteristics, reflecting a capacity to handle the natural variability inherent in biological signals, such as acoustic song production. By providing a functional parameter map for these interactions, we highlight how robust stimulus-pair association emerges from the asymmetric contributions of initiation and readout drives.

## Discussion

Temporal sensitivity for structured sequences of sensory events is a defining property of higher-order neurons across auditory, visual, and somatosensory systems.^13^^,^^22^^,^^48^^,^^49^ In the songbird auditory system, this feature is particularly striking in neurons of the HVC, which respond selectively to ordered stimulus pairs, associating their input signals even when the gap between them spans several hundreds of milliseconds.^4^ This implies that the circuitry through which these neurons operate must possess the capacity to retain information across hundred-millisecond-scale gaps following a first stimulus, while also allowing a precise millisecond-scale temporal alignment once the appropriate second stimulus arrives. This dual-timescale requirement presents a fundamental computational challenge: how can a single circuit be both broad enough to bridge long gaps and sharp enough to act as a high-precision coincidence detector?

In principle, different types of combination sensitivity might exist, and can be distinguished according to the different levels of circuit organization and the timescales over which they operate. Combination sensitivity as widely explored in the literature typically operates within a single circuit layer and relies on relatively narrow temporal windows, generally on the order of a few to several tens of milliseconds.^5^^,^^12^^,^^13^^,^^14^^,^^15^^,^^18^^,^^25^ In the auditory system, one of the best studied examples of this processing is found in the delay-tuned neurons within the inferior colliculus and related midbrain regions of echolocating bats.^3^

Foraging in bats relies on the high precision of echolocation, which is an active sensory process that is governed by strict temporal constraints.^50^ Delay-tuned neurons are an integral component of this process, acting as biological chronometers by translating the temporal interval between an emitted pulse and its returning echo into a precise calculation of target distance. These neurons show maximal facilitation responses at behaviorally relevant delays.^27^^,^^51^^,^^52^ At the circuit level, these responses arise from postsynaptic facilitation occurring at the level of the delay-tuned neuron itself. Mechanistically, inhibition triggered by the emitted pulse (the first stimulus) induces a transient hyperpolarization that primes the combination-sensitive neuron (the delay-tuned neuron). This priming induces a fundamental shift in the intrinsic membrane state of the neuron, preparing it for a subsequent excitation by the returning echo (the second stimulus), within a narrow coincidence-detection window.^3^^,^^26^^,^^27^ Notably, neural circuits wired for generating the response of delay-tuned neurons are found in brain regions early in development, even before the bat’s actual engagement with echolocation. This implies that bats have their fast-delay processing circuitry prewired and optimized for their immediate survival needs.^53^

While both bats and songbirds employ complex vocalizations, these vocal behaviors can serve different biological functions. Here, our bat comparison is restricted specifically to echolocating bats, in which biosonar signals are used for real-time navigation and survival.^52^^,^^53^ We note, however, that bats also produce complex social communication vocalizations, so the comparison developed here concerns echolocation-related temporal processing rather than bat vocal behavior more generally. Songbirds, by contrast, utilize melodious sequences as a more complex medium for social communication.^54^ Unlike the immediate navigational purpose of echolocation, songbirds must actively learn to sing their songs, discriminate between themselves and others, and to apply this knowledge within appropriate environmental contexts.^54^^,^^55^^,^^56^ Their song repertoires and neural selectivity emerge over a developmental trajectory that supports their higher-level social functions such as mate attraction, territorial signaling, and individual identification, which becomes a critical factor in their lives.^5^^,^^13^^,^^26^ Multiunit recordings of HVC neurons in developing white-crowned sparrows^57^ showed that young birds who had completed their sensory learning phase, but had not yet begun singing, lacked population-level selectivity for tutor song sequences. Instead, sequence-selective firing began to emerge during the plastic song phase, leading to the bird’s preference for its own developing vocalizations (BOS) over the tutor song.^58^^,^^59^^,^^60^ This supports the notion that temporal selectivity in the HVC is not innate but a reflection of the bird’s cumulative vocal experience.

This functional emergence parallels robust developmental changes in both intrinsic and synaptic properties. As HVC neurons mature, those that project to region X (HVC_X_ neurons) begin to show an increased voltage sag and enhanced rebound depolarization following hyperpolarization, which is indicative of strengthened H-current ($I_{H}$) and low-threshold calcium ($I_{CaT}$) conductances.^45^^,^^61^^,^^62^ This intrinsic excitability modulation,^63^ along with synaptic transformations that sharpen auditory tuning^38^ support the view that sequence sensitivity in songbirds is constructed through plasticity. Although more research is needed to confirm the exact mechanisms, current data suggest a clear departure from the bat’s model: in songbirds, the capacity for long-range temporal integration is not given from birth, but rather a skill that the circuit “learns” as it matures, which calls for a fundamental update to existing models of temporal combination sensitivity of the HVC. While *in vitro* and *in vivo* studies have established that HVC neurons can associate stimuli separated by hundreds of milliseconds,^4^^,^^5^^,^^15^^,^^18^^,^^26^ the circuit-level mechanisms supporting such extended delays have remained largely underspecified. Classical inhibition-excitation motifs discussed above, operating solely at the level of the combination-sensitive neuron, are effective for capturing the “final” tens of millisecond events for successive stimuli association, but fail once a short inter-stimulus interval is introduced (Figures 2, S2, and S3). This implies that simple postsynaptic facilitation, on its own, is insufficient to bridge the extended temporal gaps characteristic of the HVC.

This limitation motivated our central hypothesis: stimulus association across long delays requires an additional circuit-level structure upstream of the CSN, capable of preserving, transforming, and gating information about the first stimulus (S1) until the second (S2) arrives. In our computational model, this is accomplished through a layered architecture consisting of a TRDL and an intermediate feedforward inhibitory convergence circuit. Notably, while combination sensitivity does not occur at the level of these upstream motifs, their coordinated activity; however, is essential for maintaining a stimulus-specific trace over extended timescales and for shaping the temporal events necessary for later stimulus association (Figure 3A).

Our results highlight the interdependence between synaptic and intrinsic events across different network nodes. Specifically, our network dynamics support a patterned activation sequence in which neurons fire in a precise cascade from the onset of S1, through the inter-stimulus gap, until the arrival of S2 (Figure 3A). In this “division of labor”, S1 serves as the trigger that initiates this cascade; reversing the temporal order of stimuli failed to produce an association (Figure 5C). The arrival of S2, synchronized with specific disinhibitory events (Figure 3, arrow annotations) stops the activity in upstream layers and triggers a shift in the CSN’s coding state that opens the coincidence window. Interestingly, while we showed that the network functionality is preserved across a range of inhibitory and excitatory synaptic conductance values onto the CSN, the resulting spiking behavior remained largely unchanged (Figure 7B). This suggests that the network is tuned to the presence and temporal order of stimuli rather than their specific intensity.

Across our findings, balanced and precisely timed inhibition emerged as the core requirement for successful association. Beyond its classical roles in sharpening temporal tuning and regulating gain,^64^^,^^65^ the feedforward inhibitory layer in our model acted as a dynamic sculptor of the CSN’s intrinsic state. Rather than serving as a passive delay line, the convergent inhibitory drive triggered an active modulation of the CSN’s membrane potential by controlling the availability of $I_{CaT}$ and the activation of $I_{H}$ (Figures 6C and 6D), bridging the gap between the network’s temporal cascade and the cell’s intrinsic readiness. Together with the TRDL, these dynamics support our suggestion that the inter-stimulus gap is not an “idle” interval, but an active integration period (Figures 4 and 6). During this phase, the CSN is continuously conditioned for facilitation through a delay-dependent priming process. Unlike traditional models that view priming as a momentary or passive event, our findings suggest that the receptive state for facilitation is progressively constructed over a finite temporal window. As inhibition unfolds, the CSN’s intrinsic gating variables are gradually shifted toward a state that favors rebound depolarization. In our simulations, this priming peaks at delays of approximately 150–200 ms (Figure 4), coinciding with the point where the magnitudes of both $I_{H}$ and $I_{CaT}$ reach their maximum. Therefore, within this favorable temporal window, the CSN is not merely released from inhibition; it transitions into an optimal excitability state that allows the subsequent excitatory input from the second stimulus to be converted into a maximal facilitatory response.

It is noteworthy that our delay-dependent priming exhibits a bounded facilitation window (Figure 3B) that shares key qualitative features with the inter-stimulus intervals for syllable-pair association reported by Margoliash and Fortune^4^: an asymmetric rise and fall of facilitation across delays and a loss of facilitation at extended intervals, with a breakdown on the order of ∼230 ms. We emphasize, however, that this correspondence is qualitative rather than a precise quantitative match to the population-averaged physiological data. In particular, discrepancies in the exact timing and magnitude of peak facilitation in our model likely reflect the minimal nature of the underlying circuitry and the absence of additional mechanisms that can tune the effective memory trace between stimuli.

A plausible missing ingredient for instance is short-term synaptic plasticity (STP; facilitation and/or depression), which can act as a synaptic “eligibility trace” with decay constants spanning tens to hundreds of milliseconds^66^^,^^67^—precisely the regime that sets the location and width of a delay-tuning curve.^68^^,^^69^ In our framework, STP could shape delay dependence in at least three complementary ways. First, if the synapses that convey the first stimulus information into the upstream delay pathway (or within the delay pathway itself) are facilitating, then early activity elicited by the first stimulus would progressively amplify the delayed inhibitory drive arriving at the output stage, steepening the rising phase of facilitation and shifting the peak toward the biologically observed delay. Conversely, short-term depression in the same pathway would naturally enforce the falling phase by attenuating the trace as inter-stimulus intervals grow, effectively tightening the window even when network activity persists.^70^ Second, STP on the inhibitory synapses that implement priming at the output stage could directly tune the rebound mechanism: facilitation would deepen/extend hyperpolarization and increase rebound gain for intermediate delays, whereas depression would speed recovery and suppress rebound at longer intervals, producing a sharper breakdown without requiring changes to intrinsic conductances.^71^^,^^72^ Third, STP on the excitatory synapses driven by the second stimulus could implement a delay-dependent “gate” where the residual presynaptic facilitation following the first stimulus could selectively boost the effectiveness of the second input only within a limited interval, while leaving the coincidence-detection logic intact.^66^

Importantly, these STP mechanisms would not replace our core explanation—distributed maintenance of the first stimulus information and delay-dependent priming via inhibition-intrinsic interactions—but would provide an additional timescale-setting layer capable of converting a qualitative match into a quantitative one. They also generate testable predictions: the delay-tuning curve should exhibit history dependence (e.g., sensitivity to recent repetitions, baseline firing rate, or paired-pulse protocols), and pharmacological or genetic perturbations that alter presynaptic release probability should systematically shift or broaden the effective association window.^66^ Finally, because the reported biological data here^4^ are population-averaged, heterogeneity in STP parameters across synapses/cells would be expected to broaden and smooth delay tuning, potentially accounting for the mismatch between single “canonical” model neurons and the population-averaged data.^68^^,^^69^

Beyond the effective window, intrinsic variables relax back toward baseline, producing post-inhibitory recovery from priming (Figure 4A) and failure to achieve a facilitated burst. Together, these results support the conclusion that extended temporal integration and precise coincidence detection are not competing strategies but complementary operations distributed across circuit layers and timescales. Our network-level perspective on stimulus-pair association complements established spectro-temporal receptive field (STRF) models,^29^ which provide quantitative descriptions of stimulus selectivity but remain largely descriptive. By representing vocalizations as spectrograms processed through linear filters, STRF approaches abstract away the biophysical and circuit mechanisms required to bridge long temporal gaps. While these models effectively capture what neurons respond to, they do not specify how the brain integrates information over time. In reality, the input to a combination-sensitive neuron is not a spectrogram, but a structured pattern of synaptic currents shaped by network architecture and intrinsic membrane properties. Our model provides a complementary approach by implementing upstream reverberatory dynamics and feedforward inhibition. These components dynamically maintain stimulus-specific information across delays, enabling precise millisecond-scale coincidence detection at the point of readout.

Finally, our findings also show that stimulus features shape CSN responses in a principled, asymmetric manner. While S1 primarily governs the “intrinsic-buildup” necessary for priming, S2 holds the key to determining the expression or magnitude of facilitation. This architecture allows the network to accommodate a broad range of syllable durations and intensities (Figure 8) while maintaining strict sensitivity to temporal order. Such robustness is advantageous given the natural variability in song production, where syllable lengths and amplitudes vary, but their temporal relationships remain behaviorally meaningful.^73^^,^^74^^,^^75^ The fact that our model generalizes across such stimulus variations strengthens the conclusion that combination sensitivity in HVC emerges from the network’s internal dynamics rather than from the specific details of the sensory input.

From a broader perspective, HVC serves as a crucial relay center for birds that learn to sing using auditory feedback.^60^ One of its downstream targets is the lateral magnocellular nucleus of the anterior nidopallium (LMAN), a key node in the anterior forebrain pathway responsible for the acquisition and refinement of vocal behavior (Brainard and Doupe 2000) and a secondary hub for CSNs.^5^ Theoretically, because combination sensitivity is first established in HVC,^4^ the fundamental “rules” of temporal association might be essentially “authored” by the HVC before being transmitted to LMAN. In fact, LMAN neurons appear to extend these rules by moving beyond simple pairwise association. As generated outputs leave the HVC and reach the LMAN, CSNs in the latter seem to further extend this associative capacity by nonlinearly integrating different subsets of syllables, leading to significantly more complex response patterns.^5^^,^^18^ As Doupe^5^ demonstrated, these neurons often require the integration of at least three syllables to trigger a facilitative response. Crucially, LMAN units possess a sharper, history-dependent selectivity; their response to a specific combination can be dramatically modulated or even suppressed by the preceding vocal context. This suggests a more complex form of piecewise song recognition that builds upon, yet significantly reformats, the fundamental temporal integration occurring in HVC.^5^^,^^19^ The hierarchical integration of these output signals, from their initial associations in HVC to their second association within the more complex, contextual selectivity in LMAN remains a fundamental question in songbird neurobiology.^18^^,^^76^ Understanding these “first principles” of temporal association in HVC opens the door to future work exploring how these signals can be further sharpened and structured downstream to guide vocal plasticity and complex auditory perception.

In summary, we present a network-level mechanism in which extended temporal integration and postsynaptic facilitation jointly transform temporally selective auditory stimuli into combination-sensitive responses. By integrating circuit-level reverberatory dynamics with intrinsic membrane processes, our results demonstrate that combination sensitivity, when operating across multiple layers and extended timescales, cannot be explained by a single synaptic motif or coincidence detector alone. Instead, we argue that this sensitivity results from the coordinated interplay of inhibitory and excitatory pathways operating across distinct temporal scales. Ultimately, our computational model provides a possible mechanistic bridge between classical, short-range coincidence detection and the longer-timescale network computations that might underlie complex auditory processing.

### Limitations of the study

Our study is based on a biophysically realistic computational model, and its conclusions should therefore be interpreted within the scope of that framework. First, the circuit architecture was designed as a minimal mechanistic motif sufficient to reproduce long-delay combination-sensitive behavior, rather than as a literal reconstruction of anatomical cell numbers or full connectivity. Second, although the model was constrained by experimentally described intrinsic and synaptic properties, it does not yet incorporate several biological features that could further shape delay tuning and improve quantitative agreement with physiological data, including short-term synaptic plasticity, cell-to-cell heterogeneity, neuromodulatory influences, and multicompartment dendritic processing. In particular, while the model captures the key qualitative features of long-timescale stimulus-pair selectivity, it does not fully reproduce the precise delay-tuning profile reported in prior physiological work, suggesting that additional mechanisms may contribute to the exact form of the empirical response. Among these, short-term synaptic plasticity is an especially plausible candidate, as it could provide an additional timescale-setting process that sharpens, broadens, or shifts the effective association window. Third, the sensory inputs were represented as abstract stimulus-selective drives rather than explicit acoustic transformations, so the model addresses circuit mechanisms of temporal association more directly than upstream sensory encoding. Finally, the framework was not designed as a quantitative fit to any single physiological dataset, but rather as a mechanistic account of how distributed inhibitory dynamics, rebound-related intrinsic currents, and circuit timing can cooperate to support temporally selective stimulus association. These limitations notwithstanding, the model generates testable predictions and provides a foundation for future studies incorporating additional biophysical and synaptic mechanisms.

## Resource availability

### Lead contact

Requests for further information and resources should be directed to and will be fulfilled by the lead contact, Arij Daou (arij.daou@aub.edu.lb).

### Materials availability

This study did not generate new unique reagents.

### Data and code availability

• Data: This study did not generate new unique datasets. All results reported in this paper were produced from computational simulations and can be reproduced from the code provided below.
• Code: MATLAB source codes that generate every figure and supplementary figure in the paper is available on GitHub at https://github.com/daoulab/combination-sensitivity-model.
• All other items: Any additional information required to reanalyze the data reported in this paper is available from the lead contact upon request.

## Acknowledgments

This work was supported by funding from the University Research Board (URB) and the Farouk Jabr Foundation at the American University of Beirut. We also thank Mr. Malek El Itani, Strategic Brand and Web Designer (Slogan), for his assistance with the cover illustrations and graphical abstract.

## Author contributions

Conceptualization, A.D.; methodology, Z.M. and A.D.; software, Z.M. and A.D.; formal analysis, Z.M. and A.D.; investigation, Z.M. and A.D.; visualization, Z.M. and A.D.; writing – original draft, Z.M.; writing - review and editing, Z.M. and A.D.; supervision, A.D.; funding acquisition, A.D.

## Declaration of interests

The authors declare no competing interests.

## Declaration of generative AI and AI-assisted technologies in the writing process

During the preparation of this work, the author(s) did not use any AI tool.

## STAR★Methods

### Key resources table

**Table:** 

REAGENT or RESOURCE	SOURCE	IDENTIFIER
**Software and algorithms**		

MATLAB R2025a	MathWorks, Natick, MA	https://www.mathworks.com/products/matlab.html
Computational models and simulations	This paper	https://github.com/daoulab/combination-sensitivity-model

### Experimental model and study participant details

This study does not use experimental models.

### Method details

#### Computational framework and biophysical model design

Combination sensitivity is a multifaceted mechanism observed in various neurons and different regions of the brain.^4^^,^^7^^,^^12^^,^^13^^,^^24^^,^^25^^,^^77^^,^^78^^,^^79^ Understanding the neural basis of this feature necessitates an investigation of network dynamics and the intrinsic cellular properties that could potentially shape neuronal firing patterns.

To construct a mathematical model capable of illustrating the effects of intrinsic and synaptic properties, we focused on the HVC of songbirds for three reasons: (1) a subset of HVC neurons are well-known to be combination sensitive,^4^^,^^17^^,^^26^ (2) the synaptic^45^^,^^80^^,^^81^ and intrinsic^45^^,^^63^^,^^82^^,^^83^ properties of neuronal populations within the HVC nucleus are well described, and (3) the underlying neurophysiological processes governing combination sensitivity with an integrated set of excitatory/inhibitory synaptic currents and a set of inward/outward ionic currents. In other words, the excitatory – inhibitory interactions in our proposed network are modeled via biophysically realistic synaptic currents (AMPA/GABA) and the intrinsic properties that govern the neurons’ firing patterns are also modeled via biophysically realistic ionic currents that have been shown to be expressed pharmacologically in the HVC nucleus, making the underlying discussion more realistic.

We developed single-compartment conductance-based Hodgkin-Huxley-type (HH) biophysical model of HVC neurons and connected them together via biologically realistic synaptic currents. Our model included excitatory HVCx-projecting neurons (HVCx) and local inhibitory interneurons (HVC_INT_). Since combination-sensitive neurons (CSNs) are considered to be a subset of HVCx neurons,^13^^,^^26^ we modeled them as such. However, we further explored their unique properties by focusing on specific connectivity, synaptic inputs, and intrinsic membrane properties, as detailed below in the section on the desired network activity. The functional forms of activation/inactivation functions and time constants were based on previous published mathematical neural models.^45^^,^^62^^,^^84^^,^^85^^,^^86^^,^^87^^,^^88^ The parameters that were varied were the maximal conductance values of some ionic currents that vary among the various neuronal subtypes,^45^ as well as the synaptic conductances relevant to the network model. Every model neuron is represented by ordinary differential equations for the different state variables as illustrated below.

Simulations of the model neurons and network were conducted using the ode23 or ode 113 numerical integrators in MATLAB (MathWorks). The source code for these simulations will be made available online at our lab's website and on GitHub (https://github.com/daoulab/combination-sensitivity-model). For brute-force searches of parameters, we used a High-Performance Computing (HPC) resource at the American University of Beirut (AUB).

#### Model development

Our model included Hodgkin-Huxley type (Na^+^ and K^+^) currents for action potential generation,^84^ a high-threshold L-type Ca^2+^ current ($I_{CaL}$) for sustained firing,^43^^,^^45^^,^^82^ a low-threshold T-type Ca^2+^ current ($I_{CaT}$) for post-inhibitory rebound firing,^45^^,^^62^ a hyperpolarization-activated inward current ($I_{H}$) to capture the sag potential,^45^^,^^82^^,^^83^^,^^89^ and a leak current ($I_{L}$) for passive membrane properties. For interneurons, we integrated a large delayed rectifier K^+^ current conductance, allowing these neurons to undershoot the resting membrane potential as seen experimentally.^45^^,^^82^^,^^83^^,^^89^ Notably, in our model, the activation curve of $I_{H}$ has been shifted negatively relative to typical resting potentials in order to reproduce strong postinhibitory rebound dynamics. Since most neurons in our network rely heavily on postinhibitory rebound and since the extent of rebound depends on how much $I_{H}$ is activated during the hyperpolarized phase and how long it stays active during recovery, if $I_{H}$’s activation curve is made too depolarized (e.g., V_1/2_ at −80 mV) neurons would begin turning on near rest potentials and would thus become partly active most of the time. This leads to a weak rebound since no additional $I_{H}$ will be recruited by inhibition. However, having a more negative V_1/2_ (between −100 mV and −110 mV), as in the case here, leads to more open $I_{H}$ channels during inhibition. This is followed by appropriate recovery at the end of inhibition, leading to a strong inward “kick” of depolarizing currents and thus a strong rebound spike.^90^^,^^91^^,^^92^ Additionally, a small-conductance Ca^2+^-activated K^+^ current ($I_{SK}$) was added only to HVC_X_ neurons (and the CSN) to model their observed spike frequency adaptation.^45^ The membrane potential for each of the HVCx and HVC_INT_ neurons, including the CSN modeled as an HVCx neuron, obeys the following equations^45^:

$$C_{m}\frac{{dV}_{X}}{dt}=-I_{L}-I_{K}-I_{Na}-I_{CaL}-I_{CaT}-I_{SK}-I_{H}+I_{app}$$

$$C_{m}\frac{{dV}_{\mathrm{INT}}}{dt}=-I_{L}-I_{K}-I_{Na}-I_{CaL}-I_{CaT}-I_{H}+I_{app}$$

where $C_{m}$ is the membrane capacitance and $I_{app}$ is the applied current. The associated equations and parameters for each of the activation/inactivation gating variables for each ionic current are given in Daou et al.^45^ and shown below. Every single model of HVC_X_ and HVC_INT_ neuron had a total of 8 and 7 ODEs, respectively, that govern their intrinsic dynamics. The integration of each synaptic current into a model neuron introduces an additional ODE to the system governing its membrane potential, as will be shown next. The values of the fixed values are given in Tables S1 and S2.

#### Voltage-gated ionic currents

The constant-conductance leak current is

$$I_{L}=g_{L}\left(V{-V}_{L}\right)$$

The remaining voltage-gated ionic currents have voltage-dependent forms with activation or inactivation kinetics:

$$I_{K}=g_{K}n^{4}\left(V-V_{K}\right)$$

$$I_{Na}=g_{Na}m_{\infty }^{3}\left(V\right)h\left(V-V_{Na}\right)$$

$$I_{A}=g_{A\;}a_{\infty }\left(V\right)e\left(V-V_{K}\right)$$

$$I_{CaL}=g_{Ca}s_{\infty }^{2}\left(V\right)\;\frac{{Ca}_{ex}}{1-e^{\frac{2FV}{RT}}}$$

where

$$x_{\infty }\left(V\right)=\frac{1}{1+{\;e}^{\frac{V-{ϴ}_{x}}{\sigma_{x}}}},x=m,a,n,s\;\text{or}\;e$$

$$\frac{dx}{dt}=\frac{x_{\infty }\left(V\right)-V}{\tau_{x}},x=n,h\;\text{or}\;e$$

$$h_{\infty }\left(V\right)=\frac{\alpha_{h}\left(V\right)}{\alpha_{h}\left(V\right)+\beta_{h}\left(V\right)}\;\text{with}\;\alpha_{h}\left(V\right)=0.128e^{\frac{V+15}{-18}}\;\text{and}\;\beta_{h}\left(V\right)=\frac{4}{1+e^{\frac{V+27}{-5}}}$$

#### Low-voltage activated T-type calcium current ($I_{CaT}$)

The low-voltage activated T-type Ca^2+^ current is described by the Goldman-Hodgkin-Katz formula, with an expression similar to that given in Terman et al.^86^

$$I_{CaT}=g_{CaT}\;{\left(a_{T}\right)}_{\infty }^{3}\left(V\right){\left(b_{T}\right)}_{\infty }^{3}\left(r_{T}\right)\frac{{Ca}_{ex}}{1-e^{\frac{2FV}{RT}}}$$

where $g_{CaT}$ is the maximal T-type conductance and $a_{T}$ and $b_{T}$ are the activation and inactivation gating variables, respectively. The activation gating for the rapidly activating channel ($a_{T}$) is treated as instantaneous and is given by

$${a_{T}}_{\infty }\left(V\right)=\frac{1}{1+{\;e}^{\frac{V-{ϴ}_{a_{T}}}{\sigma_{a_{T}}}}}$$

The inactivation variable ($b_{T}$) is also treated as instantaneous and depends on the slowly operating variable r, which reflects the availability of the $I_{CaT}$ current. This is given by

$${b_{T}}_{\infty }\left(r_{T}\right)=\frac{1}{1+{\;e}^{\frac{r_{T}-{ϴ}_{b}}{\sigma_{b}}}}-\frac{1}{1+{\;e}^{\frac{-{ϴ}_{b}}{\sigma_{b}}}}$$

The slowly operating gating variable $r_{T}$ is governed by

$$\frac{dr_{T}}{dt}=\frac{{{\;r}_{T}}_{\infty }\left(V\right)-r_{T}}{\tau_{r_{T}}\left(V\right)}$$

where

$${r_{T}}_{\infty }\left(V\right)=\frac{1}{1+{\;e}^{\frac{V-{ϴ}_{a_{T}}}{\sigma_{a_{T}}}}}$$

and

$$\tau_{r_{T}}\left(V\right)=\tau_{r_{0}}+\frac{\tau_{r_{1}}}{1+{\;e}^{\frac{V-{ϴ}_{r_{r_{T}}}}{\sigma_{r_{r_{T}}}}}}$$

$I_{CaT}$ mediates low-threshold rebound excitability, which is essential for coincidence-dependent bursting in the CSN.

#### Calcium-dependent potassium current ($I_{SK}$)

The small conductance potassium current ($I_{SK}$) is modeled as in Daou et al.^45^ as

$$I_{SK\;}=g_{SK\;}k_{\infty }\left({\left[{Ca}^{2+}\right]}_{i}\right)\left(V-V_{K}\right)$$

where

$$k_{\infty }\left({\left[{Ca}^{2+}\right]}_{i}\right)=\frac{{\left[{Ca}^{2+}\right]}_{i}^{2}}{\;{\left[{Ca}^{2+}\right]}_{i}^{2}+k_{s}^{2}}$$

is the steady-state activation function of the SK current based on the levels of intracellular calcium. The constant $k_{s}$ is the dissociation constant of the Ca^2+^-dependent current, and ${\left[{Ca}^{2+}\right]}_{i}$ is the intracellular concentration of free Ca^2+^ ions and is governed by:

$$\frac{d{\left[{Ca}^{2+}\right]}_{i}}{dt}=-f\;\varepsilon \left(I_{CaL}+I_{CaT}\right)+k_{Ca}\left({\left[{Ca}^{2+}\right]}_{i}-b_{Ca}\right)$$

The constant f represents the fraction of free-to-total cytosolic Ca^2+^, whereas the constant ε combines the effects of buffers, cell volume, and the molar charge of calcium. Also, the constant $k_{Ca}$ is the calcium pump rate constant, and $b_{Ca}$ represents the basal level of Ca^2+^ (Table S1).

#### Hyperpolarization-activated inward current ($I_{H}$)

The hyperpolarization activated inward current’s activation is modeled as in Destexhe and Babloyantz^85^ using a fast component ($r_{f}$) and a slow component ($r_{s}$) as follows:

$$I_{H}=g_{H}{\;[k_{r}r}_{f}+\left(1-k_{r}\right){\;r}_{s}]\left(V-V_{h}\right)$$

The fast activation component is given by

$$\frac{dr_{f}}{dt}=\frac{{r_{f}}_{\infty }\left(V\right)-r_{f}}{\tau_{r_{f}}\left(V\right)}$$

where

$${r_{f}}_{\infty }\left(V\right)=\frac{1}{1+e^{\frac{V-{ϴ}_{r_{f}}}{\sigma_{r_{f}}}}}$$

and

$$\tau_{r_{f}}\left(V\right)=\frac{p_{r_{f}}}{\frac{-7.4\left(V+70\right)}{e^{\left(\frac{V+70}{-0.8}\right)}-1}+65\;e^{\left(\frac{V+56}{-23}\right)}}$$

The slow activation component obeys

$$\frac{dr_{s}}{ds}=\frac{r_{s_{\infty }}\left(V\right)-r_{s}}{\tau_{r_{s}}}$$

where

$$r_{s_{\infty }}\left(V\right)=\frac{1}{1+e^{\frac{-\left(V-{ϴ}_{r_{s}}\right)}{\sigma_{r_{s}}}}}$$

In our implementation of the H-current, the fast and slow activation components follow the two-component formalism of Destexhe et al.,^85^ adapted to match the biophysical properties of HVC neurons with (V_1/2_) in the ∼-85 to -95 mV range. Current activation remains minimal near rest (∼ -65 to -70 mV) and increases steeply over moderate hyperpolarized voltage range (∼ -75 to -80 mV), ensuring that $I_{H}$ contributes primarily during the inhibitory gap and early rebound rather than during ongoing depolarized activity.

Notably, while $I_{H}$ has been extensively characterized *in vitro* in HVC neurons and inferred *in vivo* from sag and long-lasting hyperpolarizations, we are not aware of *in vivo* voltage-clamp measurements that fully resolve its activation curve. Our implementation therefore follows the HVC-specific model of (Daou, Ross et al. 2013), calibrated to slice data, and uses these kinetics within the context of a network that confines the membrane potential to physiologically reasonable ranges.

#### Synaptic currents

To replicate the *in vivo* electrophysiological characteristics observed in HVC neurons, we integrated biologically realistic synaptic currents into the model alongside the above-described ionic currents. These synaptic currents represent excitatory (AMPA) and inhibitory (GABA_A_) synaptic inputs received by postsynaptic HVC neurons within the network. While there is a lack of experimental data on synaptic connections within HVC specifically in the context of combination sensitivity, we relied on data obtained from pharmacological and dual intracellular recordings across the broader HVC network, as described earlier.^80^^,^^81^

Each synaptic current represents the synaptic input(s) from the presynaptic cell(s) to a given postsynaptic neuron, and is modeled as $I_{syn}=\sum_{X}I_{X\to Y}$ where $I_{X\to Y}=g_{X\to Y}s_{X\to Y}\left(V-V_{X\to Y}\right)$. Here the summation is taken over the presynaptic HVC neurons where X represents a presynaptic cell, Y represents a postsynaptic cell, and $V_{X\to Y}$ is the reversal potential for the synapse in the postsynaptic cell with $V_{X\to Y}=V_{AMPA}$ for excitatory input and $V_{X\to Y}=V_{{GABA}_{A}}$ for inhibitory input.

The model equations for the synaptic currents are the following, adopted from earlier studies^93^^,^^94^:

$$I_{AMPA}=\bar{g_{AMPA}}s_{AMPA}\left(V-V_{AMPA}\right)$$

$$I_{{GABA}_{A}}=\bar{g_{{GABA}_{A}}}s_{{GABA}_{A}}\left(V-V_{{GABA}_{A}}\right)$$

where $s_{AMPA}$ and $s_{{GABA}_{A}}$ satisfy

$${\frac{d_{s}}{d_{t}}=a}_{r\;}\left[T\right]\left(1-s\right)-{\;a}_{d}s$$

Here [T] represents neurotransmitter release triggered by presynaptic depolarization, and is given by

$${\left[T\right]}_{pre}=\frac{T_{\max}}{1+\exp\left(\frac{V_{pre}-V_{T}}{K_{p}}\right)}$$

with $T_{\max}=1,K_{ps}=5,V_{T}=2$.

For GABA_A_, $a_{r}=5$ and $a_{d}=0.18$, while for AMPA, $a_{r}=1.1$ and $a_{d}=0.19$.

In our model, we incorporated only two synaptic currents: excitatory synapses (AMPA) and inhibitory synapses (GABA_A_). These AMPA receptor-mediated excitatory currents and GABA_A_ receptor-mediated inhibitory currents exhibit relatively simple activation kinetics.^45^^,^^62^ Their well-defined dynamics are governed by established principles and do not require additional, intricate parameters for fine-tuning. We thus excluded NMDA and GABA_B_ receptor-mediated synaptic currents from our models for the following reasons: Both NMDA and GABA_B_ currents introduce additional layers of complexity due to their dependence on factors such as Mg^2+^ concentration (NMDA) and G-protein dynamics (GABA_B_) (Destexhe, Rudolph et al. 2001). Modeling these dependencies would necessitate incorporating supplementary ordinary differential equations (ODEs) and associated parameters that are currently lacking for HVC neurons. Our primary objective was to capture the core dynamics of excitation and inhibition within the network. AMPA and GABA_A_ currents can represent these essential processes, enabling us to investigate the mechanisms underlying network behavior without introducing unnecessary complexity.

#### Model parameter sources: HVC physiology vs. canonical gating models

To ensure biological specificity, all intrinsic current magnitudes and firing-pattern calibrations used in our model were derived from songbird HVC physiology. In particular, maximal conductances for Na^+^, K^+^, L-type Ca^2+^, T-type Ca^2+^, HCN ($I_{H}$), and SK currents were taken from the HVC model by^45^ and constrained by *in vivo* and *in vitro* recordings from zebra finch HVC neurons.^43^^,^^45^^,^^82^^,^^83^^,^^89^^,^^95^ These values were further restricted to reproduce hallmark HVC properties such as sag responses, rebound spiking, spike-frequency adaptation, and plateau-riding bursts. Where songbird-specific kinetic equations were unavailable, we adopted standard Hodgkin-Huxley mathematical formalisms. That is, for fast Na^+^/K^+^ gates, these were adopted from Hodgkin and Huxley,^84^ $I_{H}$ gating structure from Destexhe et al.,^96^ T-type Ca^2+^ kinetics from Terman et al.,^86^ and SK activation from Terman et al.^86^ and Dunmyre et al.^88^ These activation/inactivation curves and time constants have been, however, reparametrized to match experimentally observed HVC firing behaviors. Thus, while canonical formulations provide the mathematical structure of gating variables, the physiological parameter values that govern excitability, rebound, and adaptation are strictly grounded in songbird HVC data rather than generic model neurons.

#### Stimulus presentation

While there is research reporting HVC’s afferent and efferent connectivity,^26^^,^^80^^,^^81^^,^^97^ little is known about the electrophysiological and anatomical identities of the auditory stimuli that the various classes of HVC neurons receive, their intrinsic and synaptic properties, and more importantly the mechanisms by which HVC neurons integrate this auditory information.

In our model, we assumed that when the first stimulus is presented for T ms, an A-selective neuron is active during that T ms, and we modeled this selectivity by a simple DC-pulse that keeps the A-selective neuron firing during that T ms. Under this abstraction, the first stimulus represents the functional occurrence of syllable A, while the second stimulus represents the functional occurrence of syllable B in an A – B syllable pair. We considered this a reasonable approach to stimulus modeling for two main reasons: (1) single syllable-selective neurons in the HVC are known to fire when the corresponding syllable is presented,^4^^,^^15^ i.e., when a syllable A or B is presented, we know that the underlying neuron is selective to these syllables primarily by their firing frequency^15^^,^^16^; and (2) we are interested in the underlying intrinsic and interactive network mechanisms that occur at different nodes within the network, which mainly occur after stimulus presentation. In other words, by following this approach, we were able to isolate different network and intrinsic mechanisms underlying temporal combination sensitivity.

Since the main computational aim of this study is to explore how network-level neural mechanisms shape postsynaptic responses across different layers, we have chosen to model song syllables as “simple drivers” whose function is to activate certain neurons without imposing assumptions about auditory preprocessing or spectral tuning. The DC-pulse input therefore serves to mark stimulus onset, offset, and duration, thus triggering a cascade of temporal interactions, inhibitory gating, and intrinsic membrane processes that give rise to combination-sensitive responses. Thus, whether a stimulus is modeled by a DC pulse or a more complex current waveform, as long as it causes the appropriate neuron to fire, the downstream network effects should remain effectively the same.

For default network simulations, and unless otherwise specified, we set the stimulus duration to 50 ms, and the stimulus intensity to 200 pA, which generates a firing frequency of 200 Hz. In the Results section on stimulus characteristics (Figure 8), we systematically varied both duration and intensity across broad ranges (Figure 8) to evaluate how these parameters affect network dynamics and to show that the generated behavior is not a rigid consequence of mere computation, but can rather be flexibly tuned to reflect realistic biological conditions. In fact, these variations can be thought of as temporal features (50 ms vs 100 ms syllable, etc.) and intensity features (louder vs softer syllable, corresponding to higher or lower current). Moreover, representing stimuli as DC pulses rather than acoustically detailed syllables could be advantageous in that it captures general principles of temporal combination sensitivity, and is not limited to species-specific song structure. This falls within the broad aim of our study, establishing a general framework that extends beyond a single vocal communication system.

#### Desired network activity

Our network model is built on the concept of postsynaptic priming,^18^^,^^19^^,^^20^ a specific form of a well-established postsynaptic facilitation mechanism^19^^,^^98^ in which inhibitory input enhances the neuron's responsiveness to subsequent excitatory input. While this mechanism is typically described at the single-cell level, our results demonstrate that postsynaptic facilitation is not the starting point of this neural computation but is rather the emergent outcome of a cascade of upstream interactions. In our work, we show that CSN “priming” does not arise in isolation but is constructed by the precise dynamics of the upstream circuitry. Specifically, a transient reverberatory delay loop (TRDL) and a feedforward inhibitory convergence (FFIC) pathway must engage with the appropriate temporal alignment for the CSN to go into this heightened state of excitability. Only when these presynaptic ensembles operate with the correct timing does the CSN become effectively primed for the facilitation that follows. Thus, what appears as a simple “inhibition-then-excitation” rule at the CSN is, in fact, the final expression of a multi-layered, time-structured computation performed across the network.

Thus, we suggest that this temporally restricted activity within the upstream network effectively encodes temporal information during a specific integration window, reflecting the interval between the two stimuli, as found experimentally.^4^ Successful combination sensitivity is an all-or-none phenomenon, requiring the fulfillment of specific conditions. That is, in the case of a stimulus pair AB, the CSN only responds when A precedes B. Reversed temporal order (BA) or single-stimulus presentation (S1 or S2 alone) do not elicit a response. Thus, we focused on the intrinsic and synaptic parameters and their underlying mechanisms that play key roles in meeting these conditions and in governing the selective initiation of the process, maintaining the temporal propagation of information, triggering an active association between different stimuli, and performing the combination. While our primary focus was on the scenario where the first stimulus precedes the second within a specific temporal window, we also explored other scenarios where the desired behavior was not attained.

The assessment of the network's ability to generate the desired firing behavior was done at two levels: (1) the CSN level, where the active association of stimuli occurs, acting as a coincidence detector; and (2) the upstream network level, where initial and intermediate response mechanisms driven by presynaptic neurons influence and shape CSN responses. At the CSN level, the generated behavior was considered successful if it generated a burst of 4-12 spikes within a duration not exceeding 80 ms window. This burst should be triggered by the near-simultaneous convergence of selectively tuned inputs within a brief temporal window, termed the “coincidence window,” typically on the order of few milliseconds. Critically, this response is conditional upon both the presence and precise temporal order of the two stimuli. The CSN should remain subthreshold at all times when presented with either a reversed temporal order or by only one of the stimuli.

At the presynaptic or upstream network level, specifically within the transient reverberatory delay loop, desired behavior was characterized by the circuit's ability to maintain patterned, firing for the duration of the delay. This period of sustained activity, termed the “integration window,” can span several hundred milliseconds, and should faithfully reflect observed temporal integration durations for natural syllable sequences (235 ± 73 ms for a syllable pair), as identified by Margoliash and Fortune.^4^ Critically, at the end of this temporal integration window, the collective output of the delay circuit should facilitate the active association of stimuli at the CSN level. Therefore, in addition to the CSN-level criteria described above, the network's behavior was considered “desired” if it also passed this temporal-delay test. In our model, we ensured that the CSN exhibits robust firing when the delay between the two stimuli varies within this physiologically relevant range. Across all simulations, we carefully monitored the firing behaviors of all neurons in the network, ensuring that each neuronal population exhibited its specific intrinsic properties such as spike frequency adaptation, sag potentials, rebound firing, and others.^45^^,^^62^

#### Quantification of model parameters

In parallel to network dynamics simulations, additional analyses were performed to explain the generated behavior in biophysical terms. In particular, we aimed to quantify key intrinsic currents ($I_{CaT}$) and ($I_{H}$) and measuring their contribution to successful stimulus association across temporal delay durations.

For each tested delay (50, 150, 200, and 280 ms), we simulated the full network under default parameter values (see “stimulus presentation” section), while varying for the time at which the second stimulus was delivered (when neuron B starts to fire). That is, the first stimulus occupied the 100 ms to 150 ms timescale, while the second stimulus occurred after delay durations that ranged from 50 ms to 280 ms (depending on the condition). The resulting membrane potential and gating variables for all neurons were obtained by numerically integrating the system using MATLAB’s ode23 solver, with a 0.01 ms step, as described above.

For each simulation, we extracted the CSN membrane voltage and its intrinsic gating variables, including the inactivation gate of the T-type channel ($r_{T}$) and the fast and slow activation gates of the H-current ($r_{f}$ and $r_{s}$). Using these state variables, we reconstructed the instantaneous currents using the same biophysical equations implemented in the model (see section on voltage-gated ionic currents) and represented the obtained current deflections in panel A of Figure 4. Since current expressions depended on dimensionless gating variables, their traces are represented by arbitrary units (a.u.). Thus, “20 a.u.”, for instance, means “20 units as defined by the model” and not 20 pA. Negative current deflections indicate an inward depolarizing current.

That is, for $I_{H}$, when the CSN become more negative than -30 mV (reversal potential), the gating variables of $I_{H}$ ($r_{f}$ and $r_{s}$) begin to activate. The more hyperpolarized the cell gets, the more $I_{H}$ opens, generating a larger inward current deflection and thus the $I_{H}$ trace becomes more negative, as evident in Figure 4. During the delay between S1 and S2, the CSN is in a hyperpolarized state in which $I_{H}$ ramps up. At the onset of S2, $I_{H}$ contributes to a depolarizing sag, seen as a rise in the $I_{H}$ trace towards zero or positive values. This upward current deflection defines the decay of inward current.

Along this process, $I_{H}$ and $I_{CaT}$ were quantified in temporal windows that reflect their physiological role. Specifically, $I_{H}$ activation was assessed during the hyperpolarization phase (during the temporal delay, gap window), defined as t ∈ [150 ms, 150 + delay], given that S1 onset occurs at t = 100 ms and offset occurs at t = 150 ms. In contrast, $I_{CaT}$ activation is most evident following the hyperpolarization phase and was therefore quantified over a 100 ms window beginning at the onset of S2 (during/post-B window: t ∈ [onsetB, onsetB + 100 ms]).

Peak current magnitudes were then computed by taking the absolute value of each current, making them independent of direction (inward/outward), and allowing comparison across mechanisms. We further normalized these peak current magnitudes relative to each current’s maximum value across delays and plotted them against the number of spikes generated by the CSN (Figure 4).

#### Intrinsic and synaptic conductance variations

Automated variations of model neuron conductances were performed in MATLAB to explore the parameter ranges for synaptic and intrinsic properties that maintain the network's firing behaviors and mechanisms. At this step, we aimed to assess the network's robustness and identify key parameters that enable the precise interactions and temporal alignment of neural events, as discussed in the “desired network activity” section, which are crucial for generating the desired CSN-level behavior, that is, a successful combination of stimuli.

We employed a one-at-a-time (OAT) sensitivity analysis approach,^99^ in which we assessed the effect of varying a single conductance parameter at a time, while holding all other parameters fixed, on the overall network behavior. We began from the default intrinsic and synaptic conductance values for each neuron in the network, which were used to generate the simulations and analyses presented in the results section. Subsequently, for each selected conductance (more details below), we ran the network simulation (which is similar to what we performed when exploring network dynamics, e.g., Figure 3A). However, for each simulation round, we introduced perturbations around its baseline value, either by increasing or decreasing the default value. This iterative process was continued until deviations in network behavior were observed, allowing us to delineate the minimal and maximal bounds within which each conductance could vary without compromising the desired functional output.

During this exploratory phase, we excluded parameter values that led to biophysically unrealistic properties in individual HVC neurons. Specifically, we discarded cases where HVC_X_ and HVC_INT_ neurons failed to exhibit their known characteristic features such as membrane sag, post-inhibitory rebound spiking, and depolarization-induced bursting. We also excluded values that resulted in altered spike amplitude or morphology. While we acknowledge that constraining the range of permissible conductance values inherently limits the parameter space explored, this approach was necessary to ensure that all network behaviors remained within the bounds of known biophysical realism.

Given the critical role of inhibition and post-inhibitory rebound mechanisms in enabling successful stimulus combination in our network model, we concentrated our exploratory analysis on three intrinsic conductances: the small-conductance calcium-activated potassium current ($g_{SK}$), the hyperpolarization-activated cation current ($g_{H}$), and the low-threshold T-type calcium current ($g_{CaT}$). All other intrinsic conductances were held constant throughout the simulations. Specifically, $g_{Na}$ and $g_{K}$ were fixed for each HVC neuron class based on values reported under *in vitro* current-clamp conditions.^45^ At such values, neurons would accurately reproduce spike morphologies, allowing appropriate features to prevail, such as action potential upstrokes, downstrokes, and plateau potentials. We also fixed $g_{CaL}$ (L-type calcium conductance), as adjustments to $g_{SK}$ alone are sufficient to maintain accurate fits to the observed spiking behavior.

The ranges of intrinsic conductance values that preserved network function are summarized in Table S3, and their distributions across simulations are visualized using boxplots in Figures S1A–S1C. For clarity in network schematics and simulations, we adopted the following naming convention: inhibitory interneurons within HVC_INT_ are designated with the letter “I” with subscripts used to differentiate individual neurons (e.g., $I_{1}$ for interneuron 1). Excitatory projection neurons from the HVC_X_ class are labeled as “E”, again with corresponding subscripts. Additionally, neurons with specialized functional tuning are labeled “A”, “B”, and “CSN,” where “A” and “B” denotes neurons selectively responsive to the first and second stimulus, respectively, and “CSN” denotes the combination-sensitive neuron.

Simulations were run at a 150-ms inter-stimulus delay, which maximizes temporal interactions among network components. Figure S1A represents the distribution of T-type calcium conductance values for selected neurons in the network: E_2_, E_3_, and the CSN. The reason examining T-type conductance variation only in these three neurons is its specific relevance to the modeled behavior. Since the T-type Ca^2+^ current is a significant contributor to postinhibitory rebound, only these three neurons require postinhibitory rebound for their activity depends on the T current. Notably, these values exhibit a relatively narrow range of variation, between 0.4 and 5 nS, suggesting that these neurons are sensitive to specific levels of T-type calcium current. This sensitivity is crucial for maintaining their firing properties and overall network behavior. The CSN, in particular, required a stricter range (0.4 - 0.9 nS): values above ∼0.9 nS caused unwanted rebound spiking in the case where only S2 is presented, which violates the biological condition whereby the CSN should only respond to the presentation of the pair of stimuli rather than to any one of them.

The SK conductance values exhibited a slightly broader range of variability (0.1 - 10 nS) compared to the T-type calcium conductance (Figure S1B). The most sensitive ranges were recorded for E_2_, E_3_, and CSN, which are precisely the cells that rely on post-inhibitory rebound to propagate or decode temporal information. This sensitivity is expected, as SK currents provide calcium-dependent afterhyperpolarization that regulates excitability following rebound depolarization. For these neurons, too little SK conductance leads to excessive excitability and unrealistically large rebound bursts, whereas too much SK suppresses rebound spiking altogether. Across all simulations, we retained only parameter values in which firing output fell within physiologically reasonable limits (≤12 spikes per burst). The H-current conductance values for the CSN exhibited the widest range of variability, from 2 to 30 nS (Figure S1C). This broad range reflects the importance of the H-current in shaping the dynamic behavior of the CSN. We have shown in our results section that while T-type calcium currents are crucial for initiating the burst, the H-current plays a key role in sustaining and prolonging the burst. Although both currents contribute to depolarization after hyperpolarization, T-type calcium currents are more sensitive to specific temporal patterns of input.

A similar OAT sensitivity analysis was performed for the synaptic conductance parameters, including all synaptic conductances. Each synaptic parameter was systematically varied around its default value while the others were held constant, following the same iterative procedure described for intrinsic conductances. This allowed us to determine the range of synaptic conductance values compatible with successful network function without compromising the fidelity of stimulus combination. The identified accepted ranges for synaptic conductances are summarized in Table S4, and their distributions across simulations are illustrated through boxplots in Figures S1D–S1F.

Each panel of the synaptic boxplots focused on a specific group of synapses: those activated by the arrival (onset) of the first stimulus (Figure S1D), those arriving at the CSN (Figure S1E), and those activated by the arrival of the second stimulus (Figure S1F). Interestingly, across all three groups, synaptic conductances exhibited a remarkably consistent variability range, broadly spanning 5 – 30 nS. This indicates that the network’s synaptic architecture is highly tolerant to parameter perturbations. Rather than depending on narrowly tuned synaptic strengths, our model maintains stable combination-sensitive behavior across a wide band of synaptic values. Such robustness confirms our findings that the output behavior emerges from the coordinated interaction of pathways (largely dictated by intrinsics) rather than from fine-tuned synaptic weights, reinforcing the generality and adaptability of our network design.

#### Simulation protocol and number of runs

For clarity, we report the total number of simulations used for each component of the study. All simulations were deterministic ODE integrations (MATLAB ode23), such that each data point corresponds to a single run under a unique parameter set. The three simple architectures in Figures 1A–1C were each evaluated under four timing conditions, yielding 12 simulations (Figures 2, S2, and S3). For the full TRDL + FFIC network, the delay sweep shown in Figure 3B (0 to 300 ms in ∼20 ms steps) required 16 full-network simulations. Intrinsic-current analyses (Figure 4) used 4 additional simulations at delays of 50, 150, 200, and 280 ms, while sequence-control tests (S1-only, S2-only, reversed) required three additional runs.

Intrinsic conductance manipulations included single-parameter sweeps for $g_{H}$ and $g_{CaT}$ (Figure 6; one simulation per conductance value) and two-dimensional sweeps for delay × $g_{CaT}$ and delay × $g_{H}$ (Figures 7C and 7D), requiring 160 simulations per heatmap. The intrinsic 2D $g_{CaT}$ × $g_{H}$ map (Figure 7A) required 96 simulations, and the synaptic $g_{GABA}$ × $g_{AMPA}$ map (Figure 7B) required 120 simulations. For the duration line plots (Figure 8A), we varied S1 or S2 duration over 10 values (10 - 200 ms) with one simulation per duration. For the intensity line plots (Figure 8C), S1 or S2 intensity was varied across 10 values (10 - 300 pA), again one simulation per intensity. The 2D duration and intensity heatmaps (Figures 8B and 8D) each used 10×10 parameter grids, yielding exactly 100 simulations per heatmap. The OAT robustness analysis (Figure S1, Tables S3 and S4) involved iterative perturbation of each conductance parameter until network behavior failed; the number of parameter sets evaluated for each conductance is provided in Tables S3 and S4.

### Quantification and statistical analysis

All simulations were performed in MATLAB using the ode23 solver. Because this study is based on deterministic conductance-based network simulations rather than experimental sampling, no inferential statistical tests were applied. Instead, outputs were quantified descriptively across defined parameter sets. In this manuscript, n refers to the number of independent simulation runs or the number of unique parameter combinations evaluated for a given analysis, not the number of animals, cells, biological replicates, or technical replicates. Each point in a line plot and each pixel in a heatmap corresponds to one deterministic simulation under a specific parameter condition. For Figure 3B, 16 simulations were performed across the delay sweep. For Figure 4, 4 simulations were performed for delays of 50, 150, 200, and 280 ms. For Figure 7A, 96 simulations were performed; for Figure 7B, 120 simulations were performed; and for Figures 7C and 7D, 160 simulations were performed per heatmap. For Figures 8B and 8D, 100 simulations were performed per heatmap. Exact numbers of simulations for each analysis are reported in the Methods, figure legends, and corresponding results sections. Where boxplots are used in the supplemental robustness analyses, center and spread are displayed graphically as the median and interquartile range, with whiskers defined in the figure legend. Mean, SD, SEM, and confidence intervals were not used unless explicitly stated.

### Additional resources

This study has not generated or contributed to a new website/forum and it is not part of a clinical trial.
