# Supplementary material for: Post-synaptic facilitation and network dynamics underlying stimulus-specific combination sensitivity
Source: iScience. 2026 May 27;29(6):116125. doi: 10.1016/j.isci.2026.116125 (PMC13235528; doi:10.1016/j.isci.2026.116125)
Supplement: Document S1. Figures S1–S6 and Tables S1–S4 [file mmc1.pdf]

iScience, Volume *29*

Supplemental information

Post-synaptic facilitation and network dynamics underlying stimulus-specific combination sensitivity

Zeina Merabi and Arij Daou

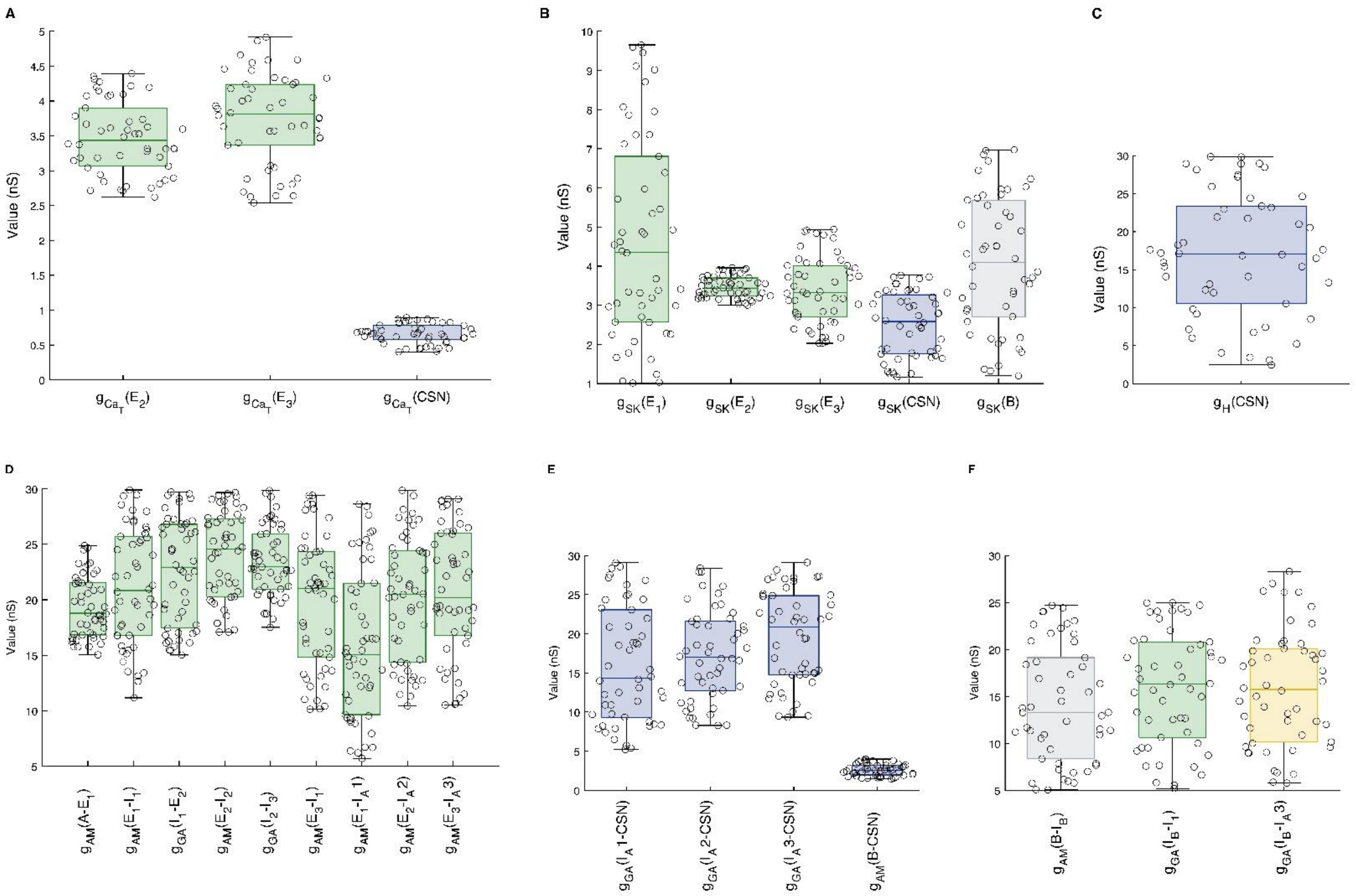

Figure S1. Distribution of identified conductance variability ranges for intrinsic ionic and synaptic currents for selected neurons from the network. The x-axis represents the neuronal population with the name of the neuron between brackets, and the y-axis represents the identified values of the sensitivity range of ionic current conductances. **(A)** Distribution of T-type calcium channel conductance (g_{CaT}) for E_2, E_3, and CSN. **(B)** Distribution of calcium-activated potassium channel conductance (g_{SK}) for E_1, E_2, E_3, B and CSN. **(C)** Distribution of hyperpolarization-activated cyclic nucleotide-modulated current conductance (g_H) for the CSN. **(D)** Boxplots representing synaptic conductance variability ranges for synaptic currents that are activated in response to stimulus 1, **(E)** arriving at the CSN **(F)** and in response to stimulus 2. Boxplots are color-coded according to their corresponding postsynaptic neuronal populations within the network.

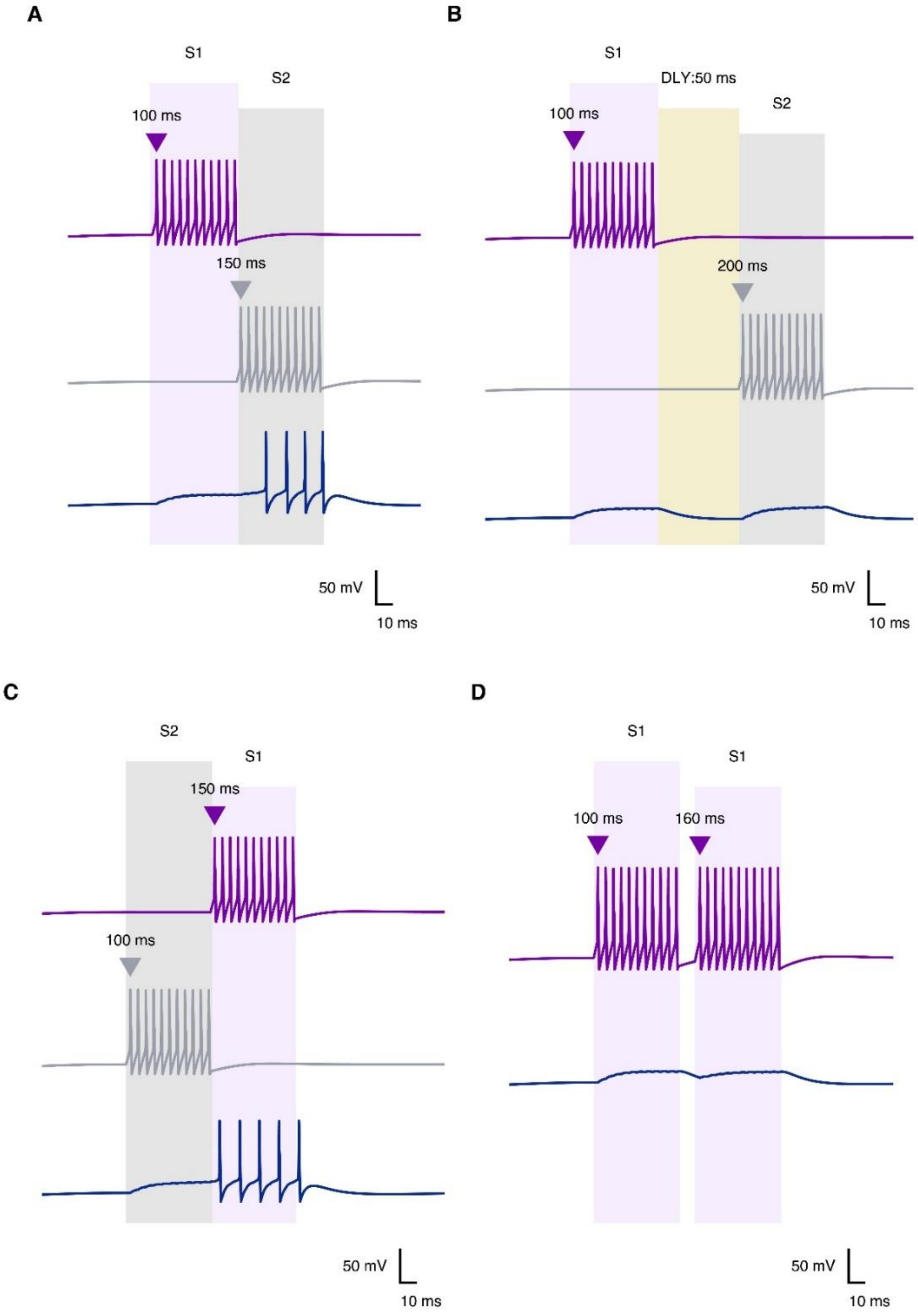

Figure S2. Simple network dynamics for the temporal summation scenario. Firing patterns for an excitatory neuron responding to the first stimulus (S1, purple trace), an excitatory neuron responding to the second stimulus (S2, gray trace) and the CSN (navy trace), shown under different conditions **(A-D)**. The onsets of S1 (purple triangle) and S2 (grey triangle) are varied across the different panels. Their respective durations are depicted by the shaded regions of the same colors. **(A)** S1 (50 ms) followed by S2 (50 ms) results in successful integration at the CSN level, generating a subthreshold increase in the membrane potential in response to S1 which then surpasses the firing threshold at the onset of S2. **(B)** A temporal delay (DLY: 50 ms, soft yellow area) results in two dissociated subthreshold increases in membrane potential at the onset of both S1 and S2, where the CSN returns to resting state at stimulus offset, thus resulting in a failure of association at the CSN level. **(C)** Reversed stimulus order generates a successful response, showing insensitivity to stimulus order. **(D)** Repeated stimulus presentation (in this case S1) fails to elevate the membrane potential above firing threshold, resulting in subthreshold responses.

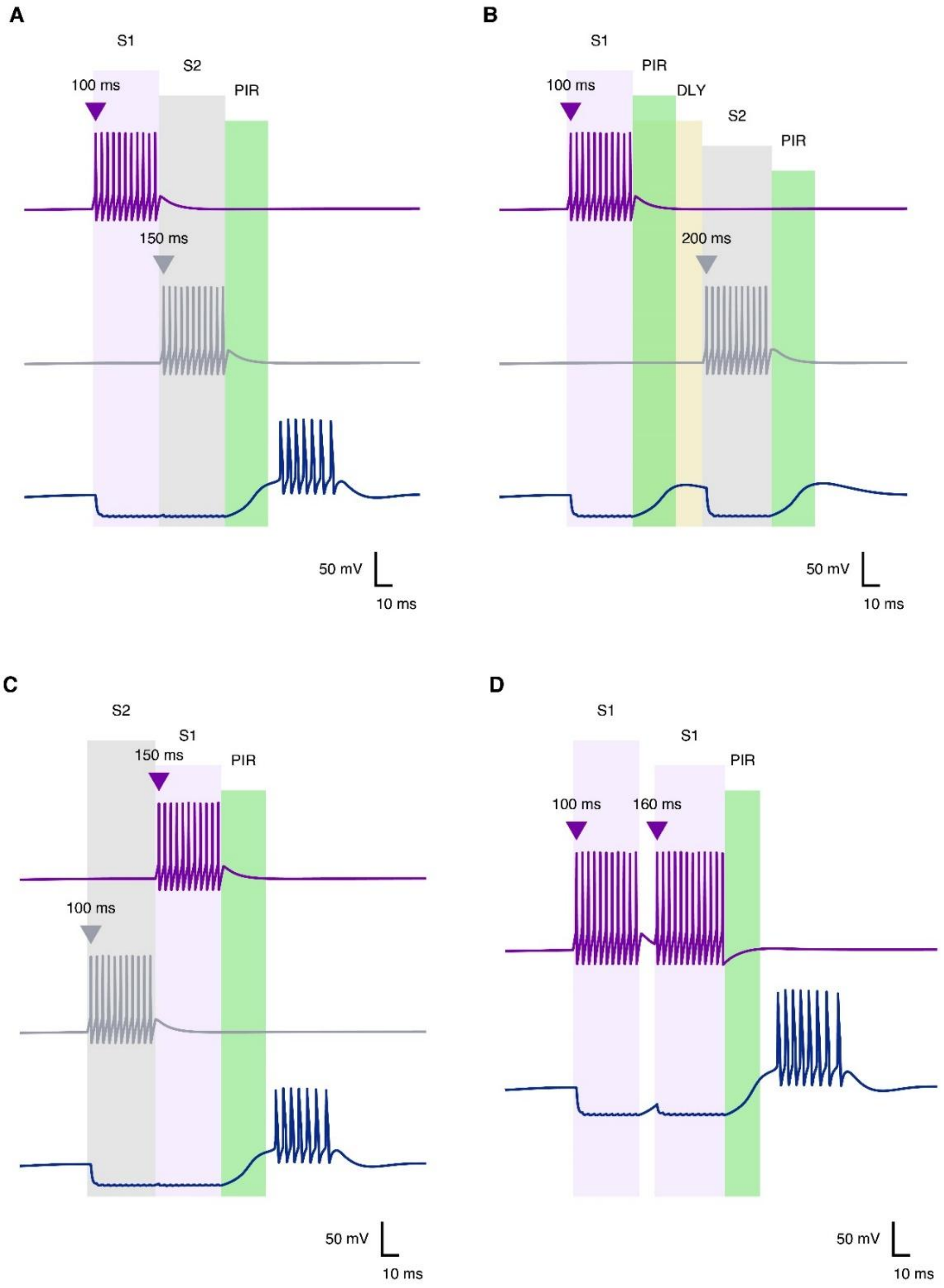

Figure S3. Simple network dynamics for the dual inhibitory scenario. Firing patterns for an inhibitory interneuron responding to the first stimulus (S1, purple trace), an inhibitory interneuron responding to the second stimulus (S2, grey trace) and the CSN (navy trace) are shown under different conditions **(A-D)**. The onsets of S1 (purple triangle) and S2 (grey triangle) are varied across the different panels. Their respective durations are depicted by the shaded regions of the same colors. **(A)** S1 (50 ms) followed immediately by S2 (50 ms) results in two successive hyperpolarizations of the CSN, the latter of which initiates a post inhibitory rebound response (PIR, green shaded area) at the offset of the S1-S2 sequence, leading to rebound depolarization and successful integration. **(B)** A temporal delay (DLY: soft yellow area, 50 ms) between the offset of S1 and the onset of S2 results in two dissociated PIR responses that do not reach firing threshold. **(C)** Reversed stimulus order and **(D)** repeated stimulus presentation (S1 in this case) result in successful response, similar to (A), showing insensitivity to stimulus order and repeated input.

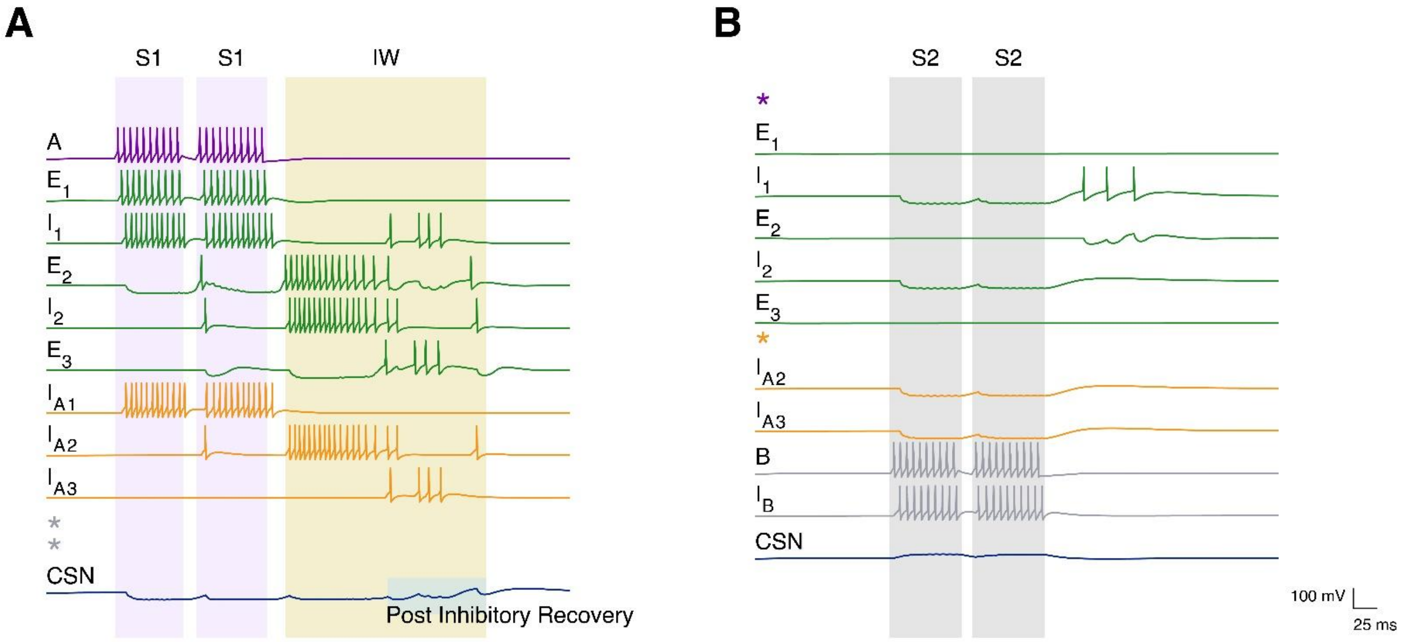

Figure S4. Stimulus-specific sensitivity of combination-sensitive neuron. **(A)** Repeated stimulus 1 presentation (S1) with a short delay (10 ms), triggers an inhibitory drive onto the CSN that stops gradually after the offset of the second S1, leading to a postinhibitory recovery, with no successful integration. Neurons of the transient reverberatory temporal delay loop (green traces) and those of the feedforward inhibitory convergence (yellow traces) show disrupted firing activity. **(B)** Similarly, repeated stimulus 2 presentation (S2) with a short delay duration (10 ms), leads to two dissociated subthreshold depolarizing events at the CSN, which return to resting state after the offset of the second S2, with no successful integration. Non-triggered voltage traces were excluded and replaced with appropriate color-coded asterisks.

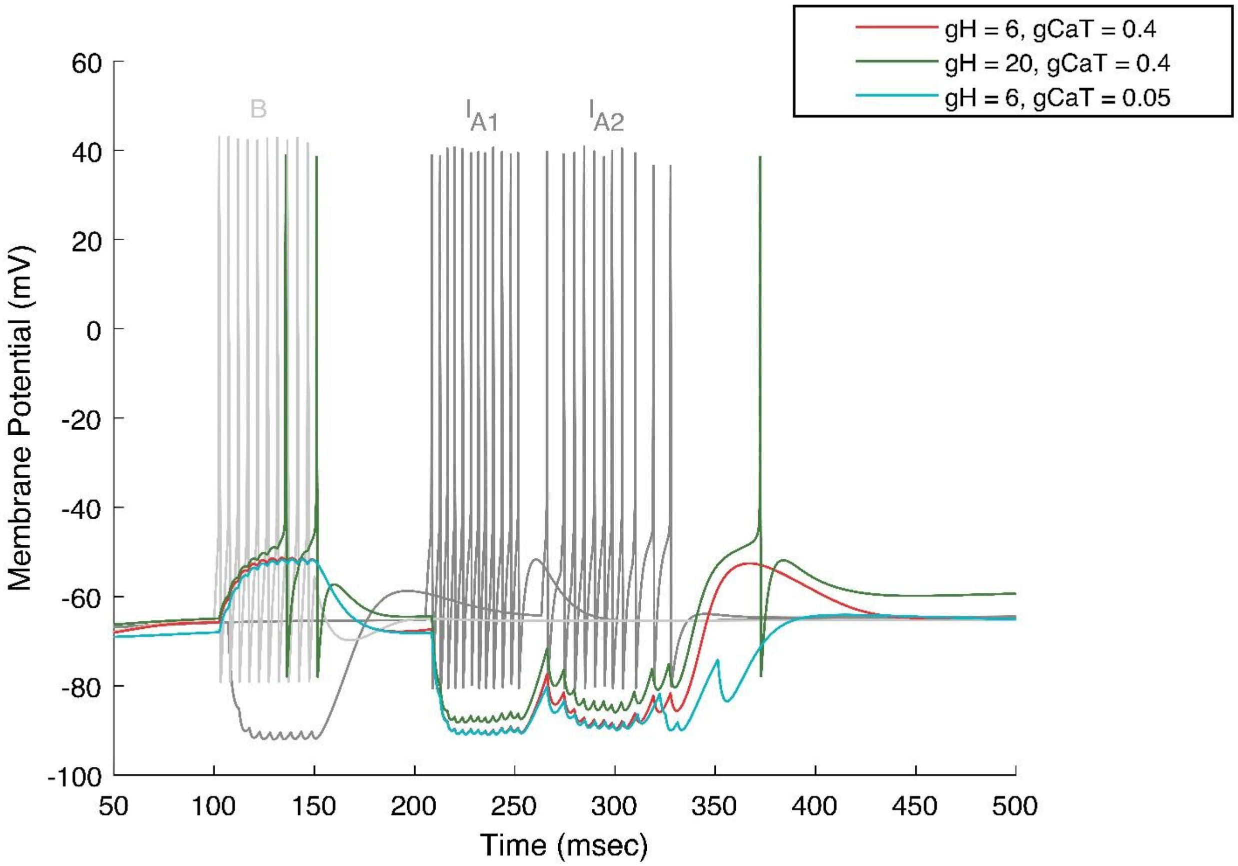

Figure S5. Reversed stimulus order abolishes successful integration at a 50 ms interstimulus delay. The figure shows firing traces of a leading B neuron (light grey trace) representing stimulus 2 (S2), followed by interneurons I_{A1} and I_{A2} (darker grey traces) representing inhibitory inputs to the CSN that are activated by the offset of stimulus 1 (S1). The CSN's firing trace is shown under two intrinsic variations in I_{Ca_T} and I_H conductances, represented by the green and blue traces, with the default trace (no variation) represented by the red trace. In all cases, successful association is not achieved, indicating loss of the temporal alignment required for facilitation.

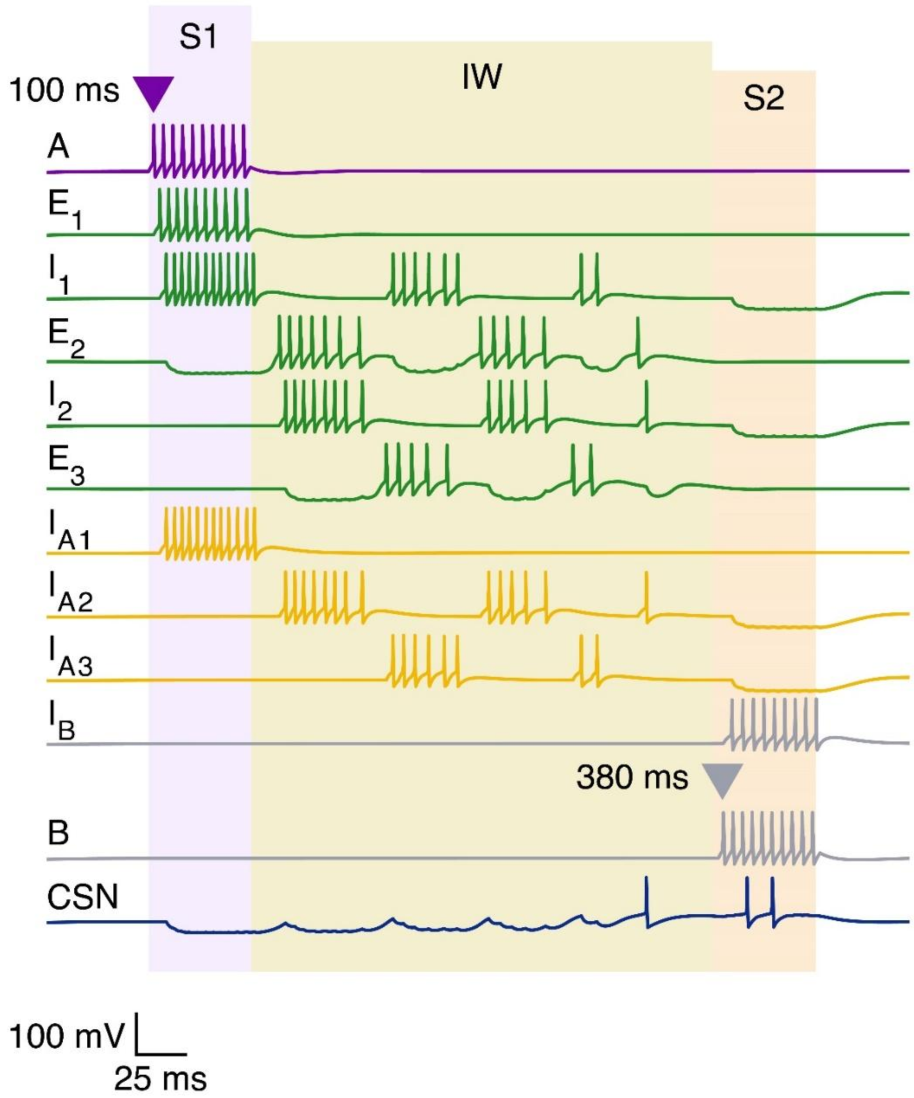

Figure S6. **Breakdown of temporal association at the upper limits of g_{Ca_T} (0.9 nS) and inter-stimulus delay (230 ms)**. While the upstream TRDL (green) and FFIC (yellow) dynamics remained intact, the attenuated inhibitory drive at this extended delay allows for a premature, weak rebound (single spike) in the CSN before the arrival of S2. Consequently, the subsequent spikes in the CSN are driven by direct excitation from neuron B rather than by facilitated association. This results in a dissociated response (3 total spikes) that fails to meet the criteria for combination-sensitive temporal alignment

Table S1. Fixed parameter values used in all simulations.

Parameter*	Value	Parameter	Value
V_L	-70 mV	V_K	-90 mV
V_{Na}	70 mV	V_h	-30 mV
g_L	2 nS	g_{Ca-L}	19 nS
g_{Na}	800 nS	τ_{r_0}	5 ms
τ_{r_1}	15 ms	τ_n	10 ms
τ_h	1 ms	τ_{r_s}	1500 ms
θ_m	-35 mV	θ_n	-30 mV
θ_s	-20 mV	θ_{r_f}	-105 mV
θ_{r_s}	-105 mV	$\theta_{r_{r_T}}$	68 mV
θ_{r_T}	-67 mV	θ_{a_T}	-85 mV
θ_b	0.4 mV	σ_m	-5 mV
σ_n	-5 mV	σ_s	-0.05 mV
σ_{r_f}	5 mV	σ_{r_s}	25 mV
σ_{r_T}	2 mV	$\sigma_{r_{r_T}}$	2 mV
σ_{a_T}	-8 mV	σ_b	-0.1 mV
f	0.1	ε	0.0015 pA^{-1} .μM. ms^{-1}
k_{Ca}	0.3 ms^{-1}	b_{Ca}	0.1 μM
k_s	0.5 μM	p_{r_f}	100

** See text for parameter definitions.*

Table S2. Parameter values that vary among neuron types.

Parameter*	HVC_x	HVC_{INT}
g_K	500 nS	1200 nS
g_h	6 nS	4 nS
V_{RMP}	-75 mV	-67 mV
C_m	20 pF	20 pF
k_r	0.3	0.01

**See text for parameter definitions.*

Table S3. Identified ranges for key neuronal intrinsic conductances in the network.

Neuron	g_{CaT} (nS)	g_{SK} (nS)	g_h (nS)
E_1	-	1 – 10	-
E_2	2.6 – 4.4	3 –4	-
E_3	2.5 – 5	2 – 5	-
B	-	1 – 7	-
CSN	0.4 – 0.9	1 – 3.8	2– 30

Table S4. Identified ranges for synaptic conductances across the network.

Synaptic Conductance values (nS)			
g_{AMPA} - Excitatory		g_{GABA} - Inhibitory	
$\mathrm{A} \rightarrow E_1$	15 – 25	$I_1 \rightarrow E_2$	15 – 30
$E_1 \rightarrow I_1$	10 – 30	$I_2 \rightarrow E_3$	17 – 30
$E_2 \rightarrow I_2$	17 – 30	$I_{A1} \rightarrow CSN$	5 – 30
$E_3 \rightarrow I_1$	10 – 30	$I_{A2} \rightarrow CSN$	8 – 30
$E_1 \rightarrow I_{A1}$	5 – 30	$I_{A3} \rightarrow CSN$	8 – 30
$E_2 \rightarrow I_{A2}$	10 – 30	$I_B \rightarrow I_1$	5 – 25
$E_3 \rightarrow I_{A3}$	10 – 30	$I_B \rightarrow I_{A3}$	5 – 30
$\mathrm{B} \rightarrow I_B$	5 – 25		
$\mathrm{B} \rightarrow CSN$	1.4 – 4		
